# Physiology and chemistry integration under UV-B across green algal *Klebsormidium* clades (Streptophyta) reveals constitutive and inducible MAA-associated photoprotective responses

**DOI:** 10.3389/fmicb.2026.1858620

**Published:** 2026-06-15

**Authors:** Barış Ballık, Fabian Hammerle, Fernanda Miyagi Pita, Mihraç Görünmek, Azam Omidi, Allegra Corelli Grappadelli, Wenxin Liang, Armin Oberosler, Kader Karsavran, Gamze Nur Çamur, Niklas Plag, Zeynep Elibol Çakmak, Klaus Herburger, Markus Ganzera, Turgay Çakmak, Ulf Karsten

**Affiliations:** 1 Department of Cell Biology of Phototrophic Marine Organisms, Institute of Biosciences, University of Rostock, Rostock, Germany; 2 Department of Molecular Biology and Genetics, Istanbul Medeniyet University, Istanbul, Türkiye; 3 Institute of Pharmacy/Pharmacognosy, Center for Chemistry and Biomedicine, University of Innsbruck, Innsbruck, Austria; 4 Faculty of Veterinary and Biological Sciences, Universidad Científica del Sur, Lima, Peru; 5 Science and Advanced Technologies Research Center, Istanbul Medeniyet University, Istanbul, Türkiye; 6 Department of Bioengineering, Istanbul Medeniyet University, Istanbul, Türkiye; 7 Department of Applied Ecology and Phycology, Institute of Biosciences, University of Rostock, Rostock, Germany; 8 Julius Kühn-Institute (JKI), Federal Research Centre for Cultivated Plants, Brunswick, Germany

**Keywords:** feature-based molecular networking, klebsormidium, mycosporine like amino acids, photoprotection, physiological stress response, streptophyte algae, UV-B (280–315 nm)

## Abstract

Stratospheric ozone variability continues to modulate biologically harmful UV-B radiation, exerting strong selective pressure on terrestrial green algae. These organisms have evolved protective mechanisms such as the biosynthesis of mycosporine-like amino acids (MAAs), which absorb UV radiation, thereby acting as sunscreen compounds. Here, we integrated ecophysiology and untargeted metabolomics to investigate UV-B-associated photoprotective response across six phylogenetically distinct terrestrial green algal *Klebsormidium* species (Streptophyta) originating from polar, alpine, and semi-arid habitats. Using a common-garden UV-B exposure approach (2 W m^−2^), we observed that UV resilience is not directly associated with total MAA content alone, but instead corresponded with contrasting patterns of MAA deployment. Alpine *K. crenulatum* maintained a constitutive MAA pool with klebsormidin A concentrations of approximately 12.4 mg^−1^ dw and exhibited only minimal MAA induction under UV-B exposure, while maintaining high photosynthetic performance with Y(II) values remaining > 0.4 and only minor reductions in growth rate (*μ*). In contrast, low-latitude species such as *K. subtile* and *K. deserticola* displayed strongly inducible MAA responses, with MAA levels increasing by approximately 120-fold under UV-B treatment, accompanied by temporary reductions in Y(II) and growth rate. UHPLC-VWD-HRMS/MS analysis followed by feature-based molecular networking identified species-specific MAA profiles beyond dominant klebsormidins, including canonical MAAs such as shinorine, asterina-330, and porphyra-334, together with additional putatively annotated UV-absorbing compounds. Polar species maintained comparatively high UV tolerance despite low MAA abundances, suggesting that additional constitutive photoprotective mechanisms might be present. Our results highlight that UV-B tolerance in *Klebsormidium* is more closely associated with MAA composition, timing, and deployment strategy than with total MAA content alone, suggesting contrasting UV-B response patterns among early-diverging streptophytes.

## Introduction

Ultraviolet-B radiation (UV-B; 280–315 nm) is an important environmental stressor that affects the survival, physiology, and distribution of phototrophic organisms. UV-B exposure is influenced by multiple environmental factors, including altitude, surface albedo, atmospheric clarity, and variability in stratospheric ozone concentrations. Although the Montreal Protocol has contributed substantially to ozone layer recovery, regional increases in UV-B exposure, particularly in polar regions affected by seasonal ozone depletion, continue to impose ecological pressure on terrestrial and aquatic ecosystems (WMO, 2022; Ding et al., 2025). In phototrophs, UV-B can damage DNA and the photosynthetic apparatus either directly or indirectly through the formation of reactive oxygen species (ROS), thereby requiring both constitutive and inducible cellular protection mechanisms (Lawrence et al., 2018; Rosic, 2019). Consequently, extreme habitats such as polar, alpine, and desert ecosystems provide valuable natural systems for investigating how closely related organisms adapt and cope with chronic UV-B stress.

In these terrestrial habitats, *Klebsormidium* (Klebsormidiophyceae), a filamentous genus of the streptophyte algae, dominates biocrusts from the poles to the Alps and deserts through a variety of metabolic strategies (Palaniappan et al., 2025), and due to its cosmopolitan distribution, high abundance, and ecological functions, it is described as an ecosystem engineer. Biocrusts are key primary producers in such ecosystems, where coverage by vascular plants is limited. As demonstrated in previous studies, *Klebsormidium* possesses a dynamic and competitive metabolic profile in response to desiccation, high irradiance, nutrient limitations, and is an excellent model for UV-B stress biology among early-diverged streptophytes (Karsten et al., 2014; Kitzing and Karsten, 2015; Futó et al., 2024). Its flexible cell walls, rich in callose, which is particularly induced by drought stress, increase its chances of survival under harsh conditions and indirectly contribute to maintaining high photosynthesis rates despite low water availability (Holzinger and Karsten, 2013; Herburger and Holzinger, 2015). In addition to these mechanical protection mechanisms, *Klebsormidium* has several cellular photoprotection properties (Rippin et al., 2018; Míguez et al., 2020).

Photoprotection in algae consists of multiple mechanisms. Although the priority and order of action of these mechanisms are still unclear, mycosporine-like amino acids (MAAs), trace amounts of phenolic compounds, carotenoids, oxidative stress control mechanisms, and DNA damage repair systems are key protective traits (Görünmek et al., 2024). MAAs possess strong absorption capacity for UV-A and UV-B wavelengths, are photostable, and do not compromise visible-light harvesting (de la Coba et al., 2019). Their biosynthetic machinery, including gadusol-related scaffolds, occurs across diverse taxa (Shick and Dunlap, 2002; Carreto and Carignan, 2011; Osborn et al., 2015; Rosic, 2019). Apart from their photoprotection properties, MAAs act as antioxidants and signal molecules, and their ecological roles or biotechnological potential can be studied in a broad perspective (Figueroa, 2021; Punchakara et al., 2023; Görünmek et al., 2025).

A recent study characterized the genus-typical MAAs klebsormidin A and B in various closely related *Klebsormidium* and *Interfilum* strains, expanding the streptophyte sunscreen repertoire (Hartmann et al., 2020). Applying experimental UVR stress showed that increased MAA accumulation coincides with moderate or minimal PSII suppression, suggesting that physiological tolerance does not scale solely with total MAA content and that intracellular composition, timing of distribution, and additional protective mechanisms may also contribute (Kitzing et al., 2014). Because MAAs comprise structurally similar and often low-abundance compounds, advanced chromatographic and mass spectrometric approaches are required for their investigation. Recent analytical developments for MAA analysis range from HPLC-DAD and CE-DAD (Hartmann et al., 2017; Orfanoudaki et al., 2020) to UHPLC-based workflows coupled with high-resolution mass spectrometry (Zwerger and Ganzera, 2022). In parallel, untargeted metabolomics approaches have increasingly been applied to characterize complex natural product mixtures (Allard et al., 2018). The GNPS (Global Natural Products Social Molecular Networking) platform provides an accessible framework for the analysis of MS/MS datasets, supporting the annotation of unknown compounds with workflows such as feature-based molecular networking (FBMN) and ion identity molecular networking (IIMN) that are capable of detecting structurally related metabolites, improving isomer resolution, and reducing data redundancy (Watrous et al., 2012; Nothias et al., 2020; Schmid et al., 2021). Combined with *in silico* annotation tools such as SIRIUS and tima (Dührkop et al., 2019; Rutz et al., 2019), these approaches have recently been adapted for the selective analysis of algal MAA metabolomes (Zwerger et al., 2023).

We therefore combined for the first time quantitative and untargeted analyses of MAAs using UHPLC-HRMS/MS and feature-based molecular networking to investigate UV-B responses across six phylogenetically distinct *Klebsormidium* species originating from polar, alpine, and semi-arid environments. The primary objective of this study was to examine whether UV-B resilience in *Klebsormidium* is more closely associated with inducible MAA accumulation or with constitutive response patterns that are not fully explained by total MAA content alone. In addition, we explored whether species-specific differences in MAA composition are associated with contrasting physiological response patterns under standardized UV-B exposure.

## Materials and methods

### Algal origin, identification, and cultivation conditions

**Figure 1:**
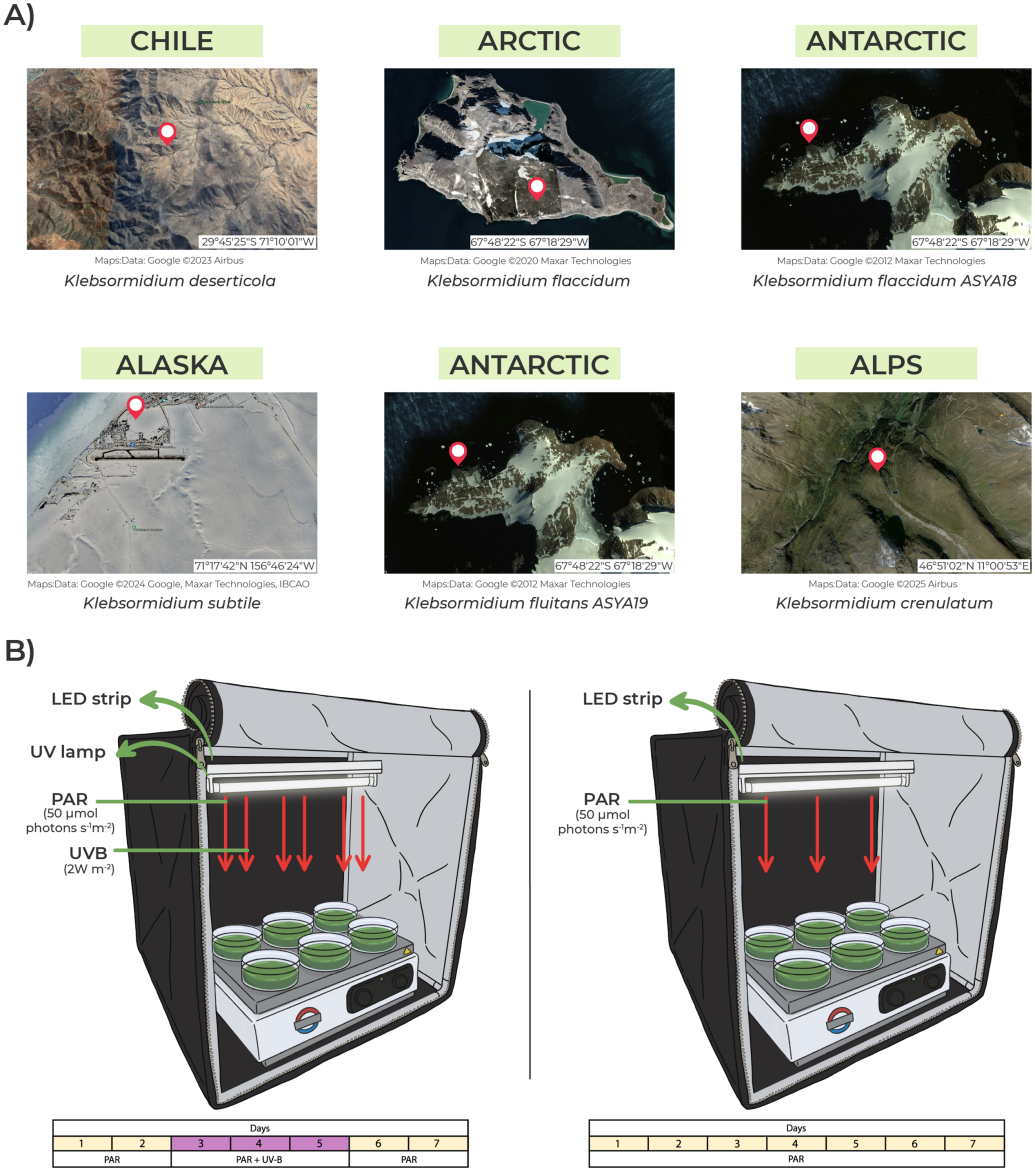
Isolation points of the *Klebsormidium* strains and a detailed explanation of the experimental setup. **(A)** Satellite views and marked isolation sites for each strain: Chile, *K. deserticola* (clade G); Arctic-Svalbard (Amsterdamøya/Danskegattet), *K. flaccidum* (CCCryo 149–01; clade B/C); Antarctica, *K. flaccidum* ASYA18 and *Klebsormidium fluitans* ASYA19 (both from lakeshore rock; clade B/C); Alaska (USA), *K. subtile* (SAG; clade E); Alps (Austria), *K. crenulatum* (SAG 2415; clade F). Pins indicate approximate collection sites. **(B)** UV-B exposure setup: schematic of the benchtop incubation tents used in this study. Left: UV-B + PAR [50 μmol photons m^−2^ s^−1^, LED strip (2500LM, 5,500 K, LED lamp beads, Germany)] condition during the induction phase, cultures in covered chambers were illuminated with constant PAR plus supplemental UV-B (2 W m^−2^, overhead UV-B lamp). Right: PAR-only condition used for the acclimation (days 0–2) and recovery (days 5–7) periods, and as a control. All incubations were run at 20 °C under a 16 h light / 8 h dark photoperiod; UV-B was applied for 72 h (days 2–5) while PAR remained constant in both treatments. Geographic images shown in panel A were generated using Google Earth Pro. Map data and imagery copyright information are provided within the respective panels.

The six *Klebsormidium* species used in this study, including their phylogenetic clades, geographic origins, habitat types, accession information, and culture collection sources, are summarized in Table 1 and Figure 1A. Antarctic isolates *K. flaccidum* ASYA18 and *K. fluitans* ASYA19 were collected from rock surfaces on Horseshoe Island during the IV Turkish National Antarctic Science Expedition in 2020. Algal material was scraped using sterile instruments and transported under near-ambient conditions to preserve viability. Preliminary identification was performed by light microscopy, followed by taxonomic confirmation according to Mikhailyuk et al. (2014). The remaining isolates representing individual species were obtained from the culture collections of the Department of Applied Ecology and Phycology, University of Rostock, SAG, and CCCryo.

**Table 1:** Overview of the six *Klebsormidium* strains used in this study, including phylogenetic clade assignment, geographic origin, habitat type, accession information, and culture collection source. Antarctic isolates *Klebsormidium flaccidum* ASYA18 and *Klebsormidium fluitans* ASYA19 were obtained during the IV Turkish National Antarctic Science Expedition and are maintained in the IMU-ASYA culture collection. Clade assignments follow previously published phylogenetic frameworks for *Klebsormidium*.

Species\strain designation	Accession	Clade	Geographic origin	Habitat/source	Culture collection
*Klebsormidium subtile* SAG 384–1	EF372517.1	E	Alaska	Snow habitat	SAG
*Klebsormidium deserticola* SG03-7.2	MH971189.1	G	Chile	Semi-arid shrub soil	University of Rostock
*Klebsormidium crenulatum* SAG 37.86	JN190354.1	F	Alps	Alpine soil crust	SAG
*Klebsormidium flaccidium* 149–01	-	B\C	Svalbard	Arctic soil crust	CCCryo
*Klebsormidium flaccidium* ASYA18	ON729294.1	B\C	Antarctica	Rock surface	IMU-ASYA
*Klebsormidium fluitans* ASYA19	ON817225.1	E	Antarctica	Rock surface	IMU-ASYA

Genomic DNA was extracted from *Klebsormidium fluitans* ASYA19 and *K. flaccidum* ASYA18 using standard protocols. Three loci were targeted: a partial 18S rRNA fragment (PCR primers 27F: 5′-AGAGTTTGATCMTGGCTCAG-3′ and 809R: 5′-GCTTCGGCACGGCTCGGGTCGATA-3′), the full ITS1 region, and a segment of rbcL2. Amplicons were Sanger-sequenced, edited (BioEdit v7.0.9), and queried against NCBI using BLASTn for taxonomic assignment. For *Klebsormidium* ASYA19, 18S rRNA showed 99.73% identity to *K. fluitans* (GenBank AM490839.1); the *Klebsormidium fluitans* ASYA19 sequence is available under GenBank ON817225. Partial rbcL2 sequences for ASYA19 and ASYA18 are deposited under ON817225.1 and ON729294.1, respectively. Strains are also maintained in the Istanbul Medeniyet University culture collection (metadata and growth conditions at: https://www.imuasya.org/collection).

All *Klebsormidium* cultures were maintained in modified 3 N-BBM liquid medium (Bischoff, 1963) at 20 °C under a 16:8 h light: dark photoperiod and an irradiance of 50 μmol photons m^−2^ s^−1^ provided by Osram Daylight Lumilux Cool White lamps (L36W/840, Osram, Munich, Germany). Cultures were initially grown in 100 mL Erlenmeyer flasks and renewed with 20% fresh medium every three weeks before transfer to 1 L bottles for biomass production under sterile-aeration.

### Clade mapping and phylogenetic tree of *Klebsormidiım* strains

The phylogenetic analysis was conducted using the Maximum Likelihood (ML) method, based on the Tamura and Nei (1993) nucleotide substitution model (Tamura and Nei, 1993). The dataset included 34 coding nucleotide sequences, covering positions 1, 2, 3, and non-coding positions, resulting in an alignment of 1,795 positions (Nei and Kumar, 2000). The resulting tree had the highest log-likelihood value (−8,541.45), and the number of substitutions per site was used to scale branch lengths (Felsenstein, 1985). The initial tree for the ML analysis was chosen as the one with the higher log-likelihood value between the Neighbor-Joining (NJ) and Maximum Parsimony (MP) trees (Saitou and Nei, 1987). To evaluate the reliability of the tree’s topology, a 500-replicate bootstrap analysis was performed, with support values displayed next to the branches (Stecher et al., 2020; Stecher et al., 2026). All phylogenetic analyses were conducted in MEGA12 using seven parallel computing threads. The final phylogenetic tree was visualized and graphically refined using the Interactive Tree of Life (iTOL) platform.

### UVR induction experiments and harvesting

Six *Klebsormidium* strains were included in a 7-day, three-phase experimental setup to assess their physiological responses to UV-B and changes in MAA accumulation and profiles. Cultures were maintained under PAR-only for the first 2 days (pre-acclimation). All strains had been maintained under controlled laboratory culture conditions for an extended period prior to the experiment and were therefore already acclimated to cultivation conditions. The 2-day pre-acclimation period before UV-B treatment was used as a short stabilization phase to ensure comparable physiological conditions and adequate growth before stress exposure, rather than as a primary environmental acclimation step. Next, they were placed under PAR + UV-B for 3 days to induce photoprotective responses, and then transferred back to PAR for 2 days to assess recovery dynamics. The PAR was set at 50 μmol photons m^−2^ s^−1^ (2500LM, 5,500 K, LED lamp beads, Germany), which is the same as normal culture conditions. The applied UV-B dose [2 W m^−2^ (G15 T8E UV-B-280-360 nm fluorescent lamp Sankyo Denki Company, Tokyo, Japan)] reflects the upper limit for MAA maximization (Lee and Shiu, 2009). UV-B irradiance was measured below the top of the culture vessels using a radiometer (Solar Light PMA2100, USA). To ensure accurate exposure values at the sample level, measurements were performed beneath a representative culture vessel lid, thereby accounting for potential attenuation caused by the lid material. All experimental groups were treated in controlled sterile chambers at 20 °C using a shaker to minimize shadow effects. Control groups were treated in PAR-only conditions for 7 days.

Harvesting for MAA identification was performed at day 5 (i.e., after 72 h of UV-B treatment). Cultures were mixed, aliquoted for chemical analyses, and dry weight was normalized. Replicate aliquots for HPLC-DAD and UHPLC-HRMS/MS were taken from the same biological cultures to ensure paired comparisons.

### Effect of UVR exposure on growth and photosynthetic performance

Growth was monitored for 7 days in transparent plates (Omnilab, Mehrfachkulturplatte, Bremen, Germany) using an *in vivo* growth fluorometer (Hansatech MFMS). Chlorophyll fluorescence was used as a proxy for biomass accumulation, allowing reliable growth detection at low biomass levels where filament aggregation of *Klebsormidium* is minimized. Chlorophyll-fluorescence was excited using 470 nm blue LEDs, and measurements were recorded with a photodiode every 24 h. For technical details see Karsten et al. (1996). Before each measurement, cells were allowed to settle to ensure consistent optical geometry (Karsten et al., 1996). The initial inoculation amount and methodological approach were modified from Gustavs et al. (2009). Initial fluorescence values were adjusted to remain within the optimal detection range of the instrument and therefore do not represent differences in biological starting conditions. Specific growth rate (*μ*) was calculated for each replicate using the equation F_t_ = F_0_e^μt,^ where F_0_ represents initial fluorescence and F_t_ represents fluorescence after t days, and values are presented as the mean of three biological replicates. The progression of raw fluorescence values over time is additionally shown in the Supplementary material to illustrate biomass development across treatments. Because fluorescence-based measurements under UV-B exposure may also reflect short-term photophysiological responses in addition to biomass accumulation, this limitation is considered when interpreting growth responses.

The effective quantum yield of PSII [Y(II)] was measured continuously during the three phases of the 7-day experiment (pre-acclimation, UV-B exposure, and recovery) using a pulse-amplitude modulated fluorimeter (PAM 2500; Heinz Walz GmbH, Effeltrich, Germany). All experiments were performed using three biological replicates (*n* = 3), with each replicate representing an independently grown culture initiated separately under identical experimental conditions rather than technical subsamples.

### MAA extraction and quantification (HPLC/DAD)

Microalgal extracts were lyophilized (CD plus-drier; Christ, Germany) and powdered. Approximately 10 mg of dried material was vortexed (Vortex-Genie 2, Scientific Industries, Inc., Bohemia, New York) with 1 mL of 90% methanol and extracted for 15 min in an ultrasonic bath at ambient temperature (Sonorex TK 52, Bandelin electronic GmbH & Co. KG, Berlin, Germany). After centrifugation at 12,500 rpm and 20 °C for 5 min (Centrifuge 5804R, Eppendorf, Hamburg, Germany), the supernatant was collected. The extraction was repeated twice under the same conditions, and the resulting supernatants were pooled. The combined extract was then evaporated to dryness under a stream of compressed air, reconstituted in 400 μL of HPLC-grade water, and filtered through cotton wool. The extracts were stored at −18 °C until being analysed.

Extracts were analyzed as described by Hartmann et al. (2020). Separation was conducted on an Agilent 1,200 HPLC system (Agilent, Waldbronn, Germany) equipped with a degasser (G1322A), a binary pump (G1312B), an autosampler (G1329B), a column oven (G1316B), and a DAD (G1315C). A Synergi Fusion RP-18 column (4 μm, 250 × 3.0 mm I. D.) protected with an RP-18 guard cartridge (20 × 4 mm I. D.; Phenomenex, Aschaffenburg, Germany) served as the stationary phase. Eluent A was a highly polar solvent mixture consisting of 2.5% aqueous methanol (v/v) plus 0.1% acetic acid (v/v) in water; eluent B was acetonitrile. The following gradient was employed: 0–15 min, 100% A; 15.1 min, 0% A; 20 min, 0% A; re-equilibration with 100% A for 10 min. Flow rate, column oven temperature, injection volume, and detection wavelength were set to 0.5 mL min-1, 30 °C, 2 μL, and 320 nm, respectively. Klebsormidin A eluted at approximately 8.90 min and klebsormidin B at 9.60 min. Because quantification was performed using porphyra-334 as a surrogate standard, reported MAA concentrations should be interpreted as semiquantitative estimates rather than absolute concentrations for structurally diverse MAAs.

The identities of the peaks corresponding to the quantified compounds, klebsormidin A and B, were confirmed by hyphenating the chromatography system to a single quad mass spectrometer from Agilent (InfinityLab LC/MSD) via an API-ES spray chamber. Drying gas flow, nebulizer pressure, and drying gas temperature were adjusted to 12.0 L min-1, 35 psig, and 350 °C. The capillary voltage for the positive ionization mode was 3,000 V. The MSD signal settings were as follows: mode, scan; mass range, 100–1,000; fragmentor, 70; gain, 1; threshold, 150; step size, 0.10; speed, 867 u sec^−1^; general: peak width = 0.1 min, cycle time = 1.06 s cycle^−1^. Extracted ion chromatograms (XICs) were generated for klebsormidin A (m/z 467–469) and klebsormidin B (m/z 305–307).

### UHPLC-VWD-HR-MS/MS analysis and feature-based molecular networking (FBMN)

Analyses of extracts and blanks (ultra-pure water) were performed according to the methods described by Zwerger et al. (2023) and Hammerle et al. (2025), with minor modifications. Separations were conducted on a Vanquish system (Thermo Scientific, Waltham, USA) consisting of a binary pump (VC-P10-A), an autosampler (VC-A12-A), a column oven (VC-C10-A), and a variable wavelength detector (VC-D40-A) connected to a Thermo Scientific Exploris 120 Orbitrap HRMS unit. A Luna Omega C18 100 Å column (100 mm × 2.1 mm; particle size 1.6 μm; Phenomenex, Torrance, USA), protected by a SecurityGuard ULTRA guard C18 pre-column, served as the stationary phase. The mobile phase comprised water with 0.25% formic acid and 20 mM ammonium formate (A) and acetonitrile (B). The applied gradient was as follows: 0 min, 0% B; 10 min, 5% B; 11 min, 90% B; 13 min, 90% B. Finally, the column was re-equilibrated with the original solvent composition (i.e., 0% B) for 20 min, which corresponds to a total run time of 33 min. The flow rate, column oven temperature, auto-sampler temperature, and injection volume were adjusted to 0.3 mL min^−1^, 17 °C, 20 °C, and 1 μL, respectively. The detection wavelengths were set to 330 and 350 nm, the data collection rate to 2.0 Hz, the response time to 2.0 s, and the peak width to 0.2 min.

The system was controlled by the Thermo Scientific Xcalibur 4.4 software. Calibration of the mass analyzer was done via the Thermo Scientific proprietary calibration mix and the respective automatic calibration function. The mass spectrometric parameters were as follows: heated-ESI ionization source, static spray voltage (positive: 3500 V), sheath gas (N_2_): 30 arbitrary units, auxiliary gas (N_2_): 17 arbitrary units, sweep gas (N_2_): 0 arbitrary units. The temperature of the ion transfer tube and vaporizer was adjusted to 370 °C and 420 °C, respectively. MS data (range 70–1,000 *m/z*) were recorded from 0 to 20 min with a resolution of 60,000 FWHM for MS^1^. The RF lens parameter was set to 70%. Data-dependent experiments were conducted with stepped collision energy mode and normalized collision energy type using HCD collision energies of 15, 30, and 45% at a resolution of 15,000 FWHM. The number of dependent scans was set to 3. The following selection of filters was employed: intensity threshold filter (1.0E5), dynamic exclusion (auto), isotope exclusion (assigned), charge state (perform dependent scans on singly charged precursors only), and apex filter (desired apex window: 75%). In addition, a specific exclusion list was created for the measurement using HPLC-grade water as a background extract with an IODA Mass Spec notebook (Zuo et al., 2021).

Raw data were converted using MSConvert (Chambers et al., 2012) and subsequently processed in MZmine 4.3.0 (Mzio GmbH, Bremen, Germany) (Schmid et al., 2023) before submission to the feature-based molecular networking (FBMN) workflow on GNPS2 (https://gnps2.org/homepage; https://gnps2.org/status?task=aa3139f533744e578c11464ecb169982#) for molecular network creation (Nothias et al., 2020). The molecular networks were visualized using Cytoscape software (Shannon et al., 2003). Metabolite annotation was performed with SIRIUS 6.1.1 (Dührkop et al., 2015, 2019, 2021). Detailed parameters for data processing and annotation are provided in the Supplementary material.

Detailed parameters for data processing and annotation, as well as the annotation levels of the (tentatively) identified metabolites, are provided in the Supplementary material. Briefly, annotations were derived from a broad, multi-layered workflow that integrated results from the FBMN workflow, compound class and individual compound annotation using SIRIUS in combination with the combinatorial MAA database, and the inclusion of characteristic UV absorption features through the applied MAA filter variable. In parallel, the identities of klebsormidin A and B were verified using the established analytical method described by Hartmann et al. (2020) supported by matching UV and fragment spectra with literature data.

### Statistical analysis

All analyses used three biological replicates per species and condition (*n* = 3) unless stated otherwise. Replicate values were processed first; summary values are reported as mean ± SD. Analyses were performed in R (v4.3.1), SPSS (v29), and Excel (v16.103.4).

Growth: Raw chlorophyll-fluorescence readings (a.u.) were recorded daily (days 0–7). Instantaneous daily growth rates were calculated per replicate as μt = ln(Ft − 1Ft) d^−1^, and summarized as growth-rate trajectories. In addition, a window-based specific growth rate over the 0–5 d interval (to the end of the UV-B phase) was computed as μ0 → 5 = 5ln(F5/F0) d^−1^. Within each species, Welch’s t-tests compared control with UVR-treated samples at day-5 μ0 → 5. Across species (within each light treatment), one-way ANOVA on μ0 → 5 tested among-species differences, followed by Tukey’s HSD for post-hoc pairwise comparisons. ANOVA assumptions were checked by Shapiro–Wilk (normality of residuals) and Levene’s test (homogeneity). If assumptions were violated, we report Welch’s ANOVA with Games-Howell post-hoc. Effect sizes are given as Cohen’s d (t-tests) and η^2^ (ANOVA). Significance threshold *α* = 0.05 (two-tailed).

Y(II): The Effective quantum yield of PS II(YII) was measured daily by PAM. For within-species Control vs. UVR contrasts at day-5, we used Welch’s t-tests. Among-species comparisons under each light treatment was done by one-way ANOVA + Tukey’s HSD with the same assumption checks and effect size reporting as above. Where time-series trends are displayed, error bars represent propagated SD of replicate means per day.

Cross-metric correlations (day-5): To integrate physiology with chemistry, we assessed monotonic associations among Δμ, ΔY(II), ΔMAA, and FC/MAA across the six species using Spearman’s rank correlation (*ρ*). Because multiple correlations were evaluated, Holm–Bonferroni adjustment controlled the family-wise error rate (*α* = 0.05). Where helpful, 95% CIs for *ρ* were obtained by a 10,000 iteration nonparametric bootstrap.

## Results

### Phylogenetic and morphological placement across *Klebsormidium* clades

**Figure 2:**
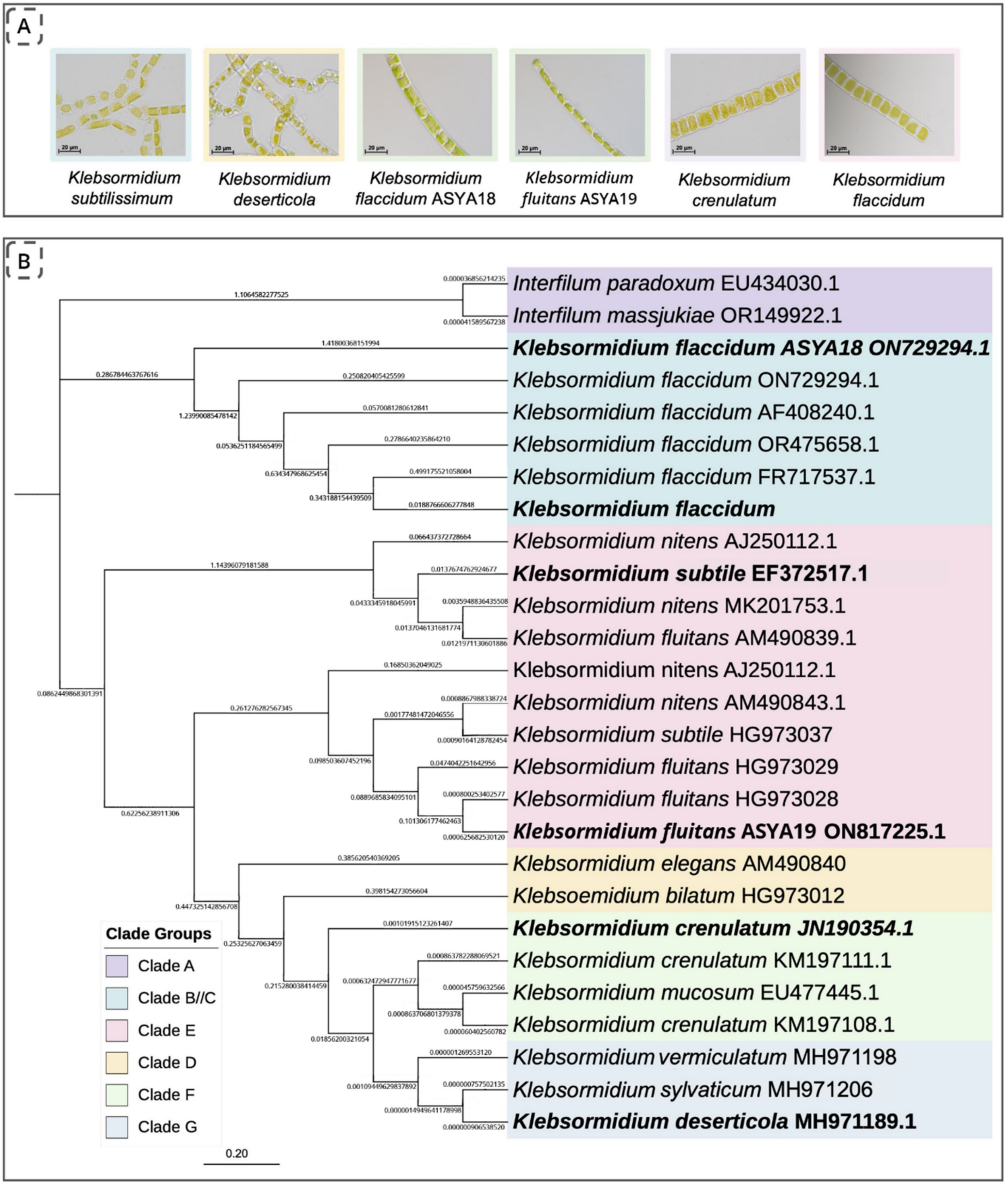
Morphological features, geographic origins, and phylogenetic relationships of six *Klebsormidium* species. **(A)** Light microscopy images of six representative species, **(B)** Maximum Likelihood phylogeny based on 1,795 nucleotide positions; bootstrap values shown for supported nodes; clades **(A–F)** are shaded, and study species are in bold.

A maximum likelihood phylogenetic analysis based on rbcL2/ITS1 marker sequences placed the six *Klebsormidium* species used in the study within four distinct clades and demonstrated that these species broadly represent the phylogenetic range of the genus (Figure 2B). *K. flaccidum* (CCCryo 149–01) and *K. flaccidum* ASYA18, together with other *K. flaccidum* accessions exhibiting a wide geographical distribution, clustered within the B/C clade with strong branch support. *K. subtile* and *Klebsormidium fluitans* ASYA19 were assigned to clade E, which includes common terrestrial species such as *K. nitens* and *K. fluitans*; light microscopy images confirmed that these species exhibit morphological features consistent with their typical long and flexible filamentous structure (Figure 2A). *K. crenulatum* is placed in clade F alongside alpine-origin species, whilst *K. deserticola* is situated in clade G, which predominantly contains species from arid regions. This phylogenetic placement reflects the ecological diversity of the examined species, which encompass polar, alpine, and semi-arid habitats, and provides a basis for evaluating interspecific differences in UV-B-associated photoprotective responses within a comparative evolutionary framework.

### Growth responses to UV-B: trajectories and specific rates

**Figure 3:**
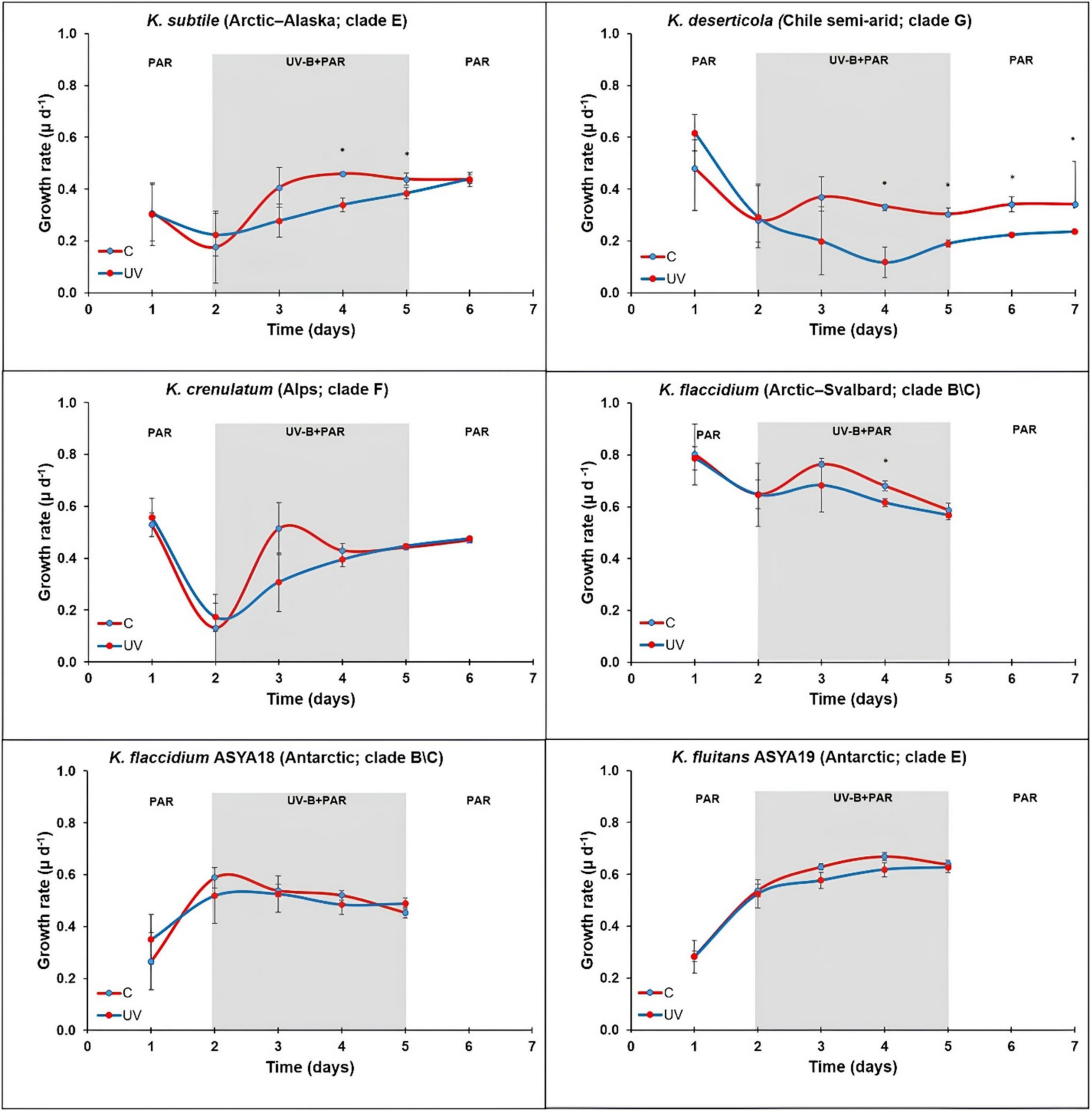
Growth rates of six *Klebsormidium* species under PAR and UV-B exposure. Time-resolved growth rates (*μ*, d^−1^) from day 0–7 for each species cultivated under common-garden conditions (50 μmol photons m^−2^ s^−1^ PAR, 20 °C). The shaded area indicates the UV-B exposure period (days 2–5; 2 W m^−2^ UV-B + PAR), while flanking periods represent PAR-only conditions. Red lines denote control cultures (PAR only), and blue lines indicate UV-B-treated cultures. Growth rates were calculated for each replicate as *μ* = ln (F_t_/F_t–₁_), where F represents chlorophyll fluorescence, and then averaged across independent biological replicates (*n* = 3; points = mean ± SD). Asterisks indicate significant differences between control and UV-B treatments at the respective time points.

The effect of UV-B on the growth of the selected *Klebsormidium* species was measured for 7 days using a three-phase regime (Figure 3) (some species reached the detection limit already on the 5th or 6th day). The six *Klebsormidium* species showed different growth rates during the first 2 days in the control condition (20 °C, 50 μmol photons m^−2^ s^−1^). In this pre-acclimation phase, *K. flaccidum* exhibited the highest growth rate, 0.648 (control) and 0.647 μd^−1^ (UVR), while the lowest values were found for *K. crenulatum* (0.131 and 0.172 μd^−1^).

During UV-B exposure (starting at day 2), some *Klebsormidium* species showed differences between the control and UV-B treatment groups. During UVR treatment (between days 3 and 5), *K. deserticola* (Chile, semi-arid) displayed a significant decrease (0.20–0.19 μd^−1^) compared to the control group (0.37–0.30 μd^−1^). It is also the most significant difference compared to all the other species on the 5th day (last day of 72 h UV-B treatment). The growth rate of *K. subtile* (Alaska) treated with UV-B was significantly suppressed (0.27 μd^−1^) compared to the control group (0.38 μd^−1^), even after 24 h of UV-B application on day 3, but the growth recovery recorded on day 6 completely matched the same growth rate of the control group in the days following treatment. For *K. subtile* and *K. deserticola* species, UV-B–treated samples showed lower growth rates than the controls throughout the 72-h UV-B exposure period, despite the differences in effects observed over time. On the other hand, the other species were minimally impacted in their growth rate and recovered to control levels during post-treatment. In *K. crenulatum* (Alps), the response differed slightly: growth was strongly reduced during the first 24 h of exposure (0.30 μd^−1^), but then stabilized at approximately 0.44 μd^−1^ by day 5. In the other three polar species (*K. flaccidum* Arctic, *K. flaccidum* ASYA18 Antarctic, and *K. fluitans* ASYA19 Antarctic), continuous UV-B exposure did not cause significant differences compared with the controls, and growth rates were only minimally affected over time.

**Figure 4:**
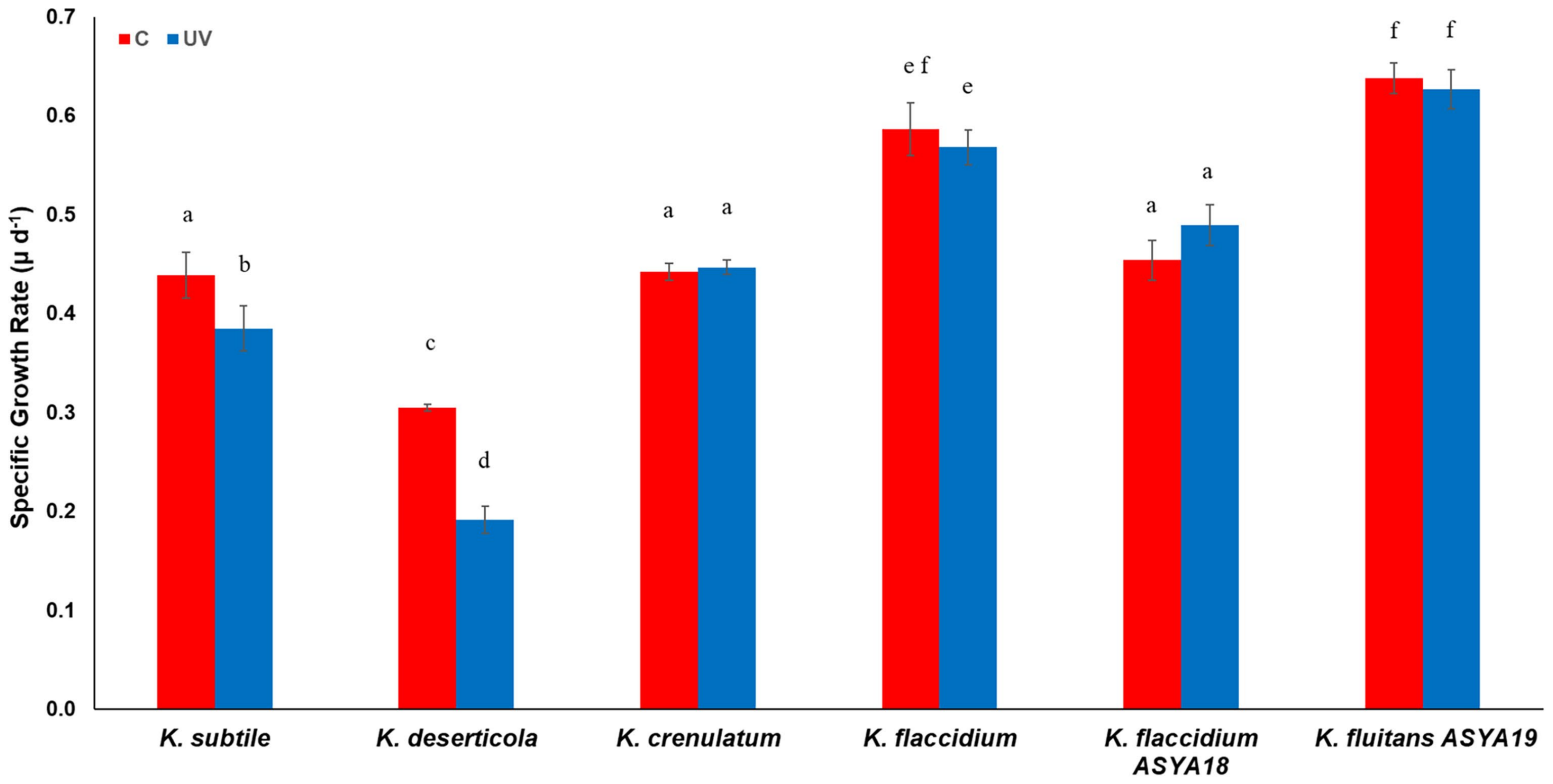
Specific growth rates (*μ*) of six *Klebsormidium* species under control and UV-B conditions. Bars represent mean ± SD (*n* = 3) of specific growth rates calculated over the 0–5 day period. Red bars indicate control (PAR only), and blue bars indicate UV-B-treated cultures. Lower-case letters denote statistically homogeneous groups based on one-way ANOVA followed by Tukey’s HSD test (*α* = 0.05); different letters indicate significant differences among treatments and species.

Across the six *Klebsormidium* species, the 0 → 5 d specific growth rate ranged from 0.19 to 0.63 d^−1^ after 72 h UV-B exposure. The highest growth rate was determined in *Klebsormidium fluitans* ASYA19 with 0.63 d^−1^, followed by *K. flaccidum* with 0.57 d^−1^, *K. flaccidum* ASYA18 with 0.49 d^−1^, and *K. crenulatum* with 0.42 d^−1^ in the treatment group, while *K. deserticola* was identified as the species with the slowest growth rate at 0.19 d^−1^ (Figure 4). The largest inhibition by UV-B was observed in *K. deserticola* (from 0.30 to 0.19 d^−1^; −36.7%) (*p* < 0.001). A clear decline was also detected in *K. subtile* (0.44 → 0.38 d^−1^; −13.6%) (*p* < 0.05). In the other four species, although a slight suppression in growth was observed at the end of UV-B exposure, no significant difference was found.

### Photosystem II efficiency under UV-B

Time-resolved Y(II) values for control (PAR only; red) and UV-B-treated cultures (blue) over 7 days. The shaded area indicates the UV-B exposure period (days 2–5; 2 W m^−2^ UV-B superimposed on 50 μmol photons m^−2^ s^−1^ PAR), while flanking periods represent PAR-only pre-acclimation (days 0–2) and recovery (days 5–7). Points represent mean ± SD (*n* = 3 independent biological replicates), measured at the same time of day using a PAM fluorometer with constant pulse settings. Asterisks indicate significant differences between control and UV-B-treated cultures at the respective time points.

To complement growth data, the PSII efficiency Y (II) was recorded during the 7-day experiment. Control samples showed minor variations over time with values between 0.5 and 0.65. UV-B exposure had no major effects on the Y(II) of the six species. The response of the *K. subtile* treatment group decreased slightly during UV-B treatment, stabilizing at 0.3, and continued to decrease on day 6 (only PAR). For this species, recovery occurred on day 7 with a Y(II) value of 0.42. Despite a major recovery, values remained significantly lower than in the control group.

**Figure 5:**
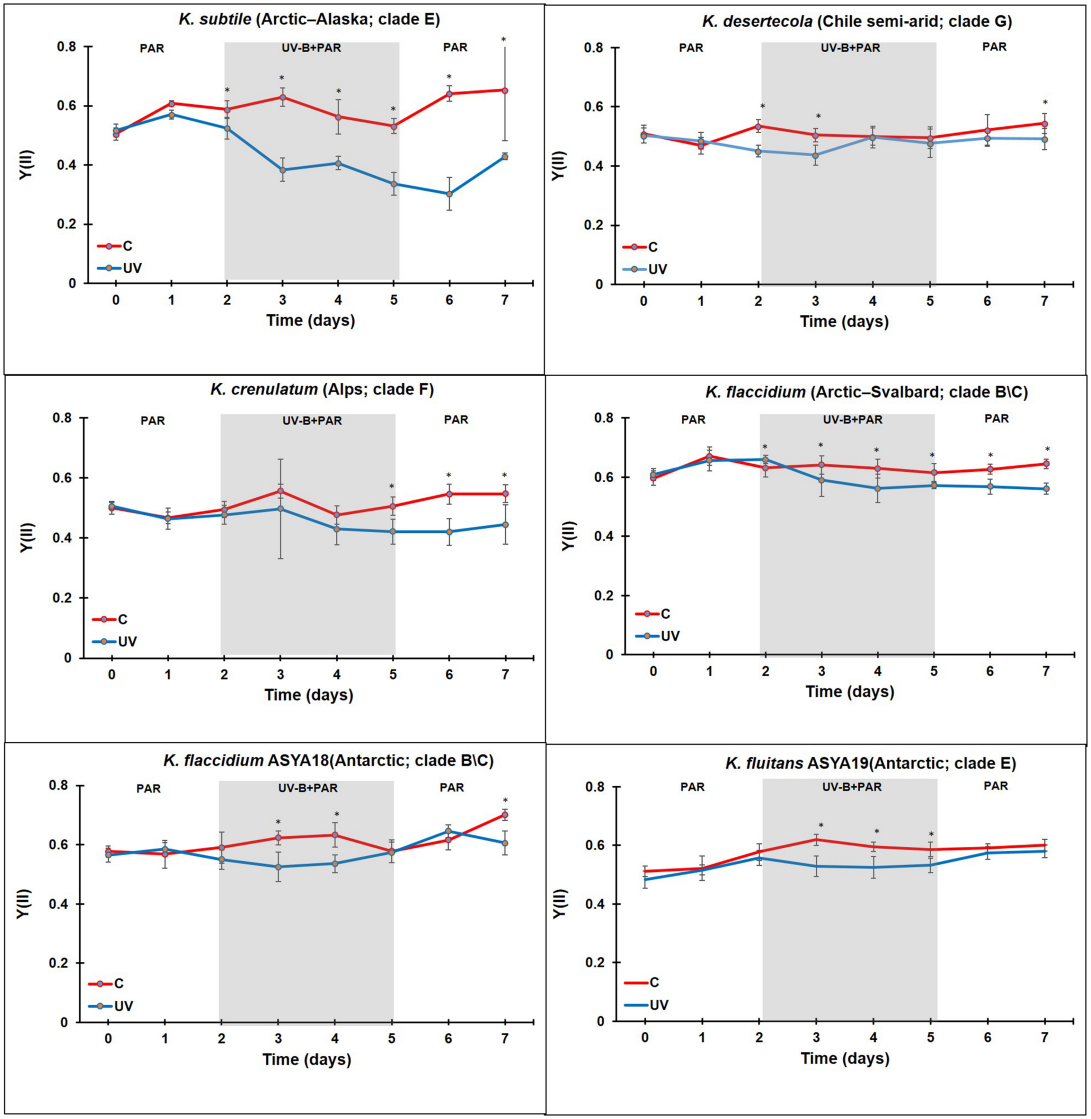
PSII performance of six *Klebsormidium* species during UV-B exposure and recovery.

**Figure 6:**
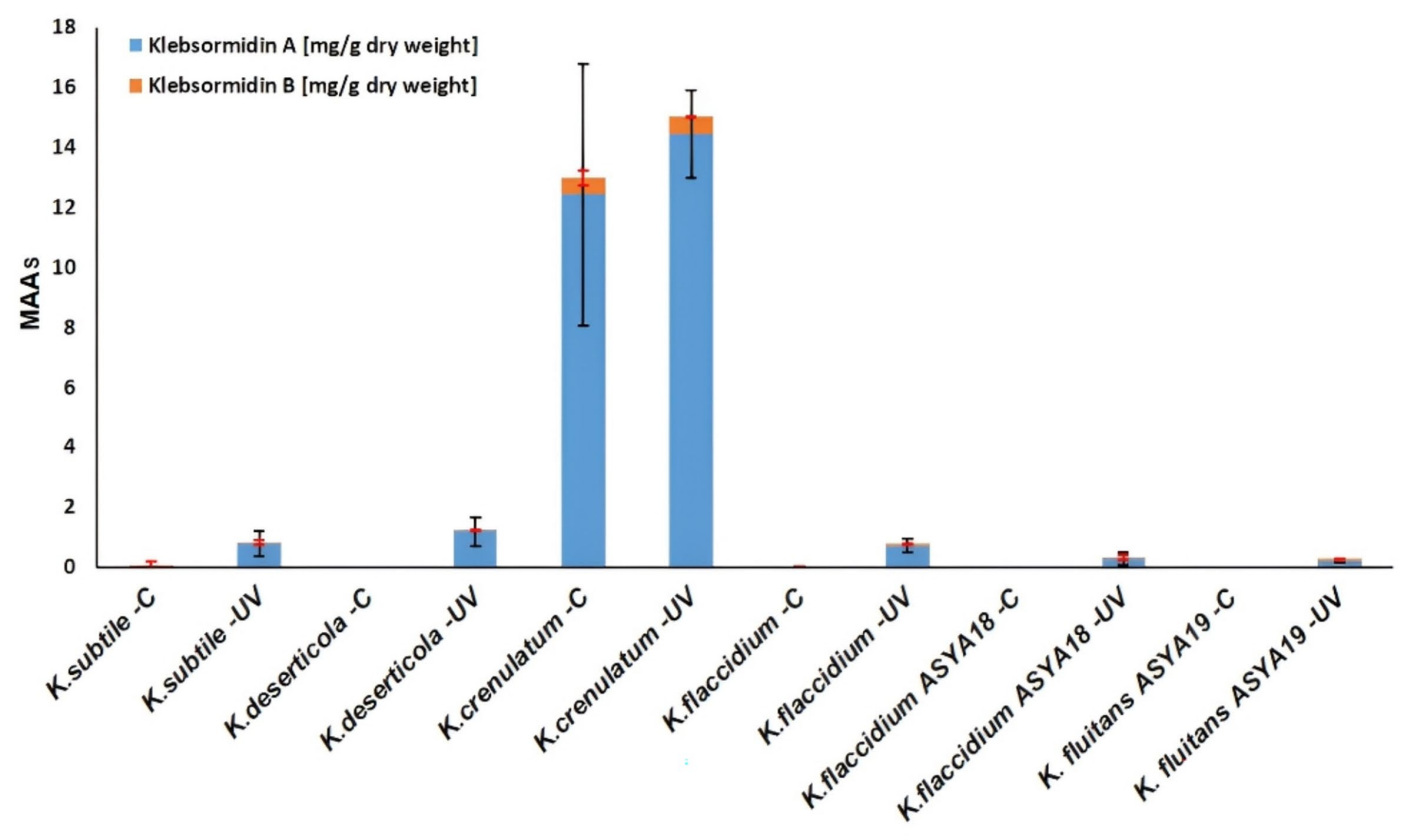
Estimates of klebsormidin A and B content across six *Klebsormidium* species under control conditions versus UV-B.

72-h UV-B exposure impacted *K. subtile* (Arctic), which exhibited a significant loss of photosynthetic yield of 0.3 compared to its control group and to the rest of the species. On the opposite, *K.* sp. ASYA 19 (Antarctic) revealed a significant yield conservation compared to all the species, apart from *K. deserticola* (Chile, semi-arid), which also showed strong invariance (Figures 4–6).

### Constitutive and inducible MAA accumulation across clades

**Figure 7:**
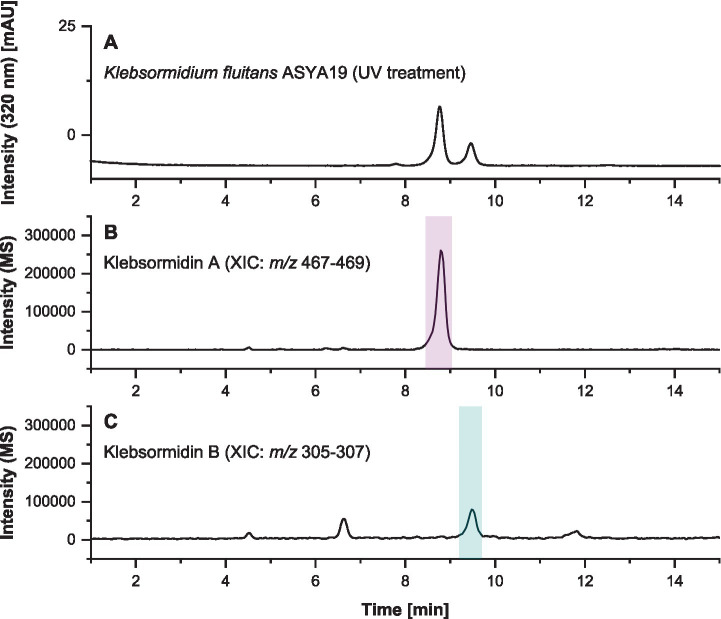
Chromatographic confirmation of klebsormidin A and B in *Klebsormidium fluitans* ASYA19 (UV-B treatment). **(A)** HPLC–DAD chromatogram recorded at 320 nm showing the MAA region of the extract. **(B)** Extracted-ion chromatogram (XIC) for klebsormidin A [integration window *m/z* 467–469, (M + H)^+^], and **(C)** XIC for klebsormidin B [*m/z* 305–307, (M + H)^+^]. Colored boxes mark the retention time integration windows used for quantification. Retention times of the highlighted peaks co-eluted with authentic standards analyzed under identical conditions, and identities were supported by UV-B spectra and MS/MS, as detailed in Methods. Intensity units in **(A)** are mAU; in **(B,C)**, they are arbitrary MS counts.

**Figure 8:**
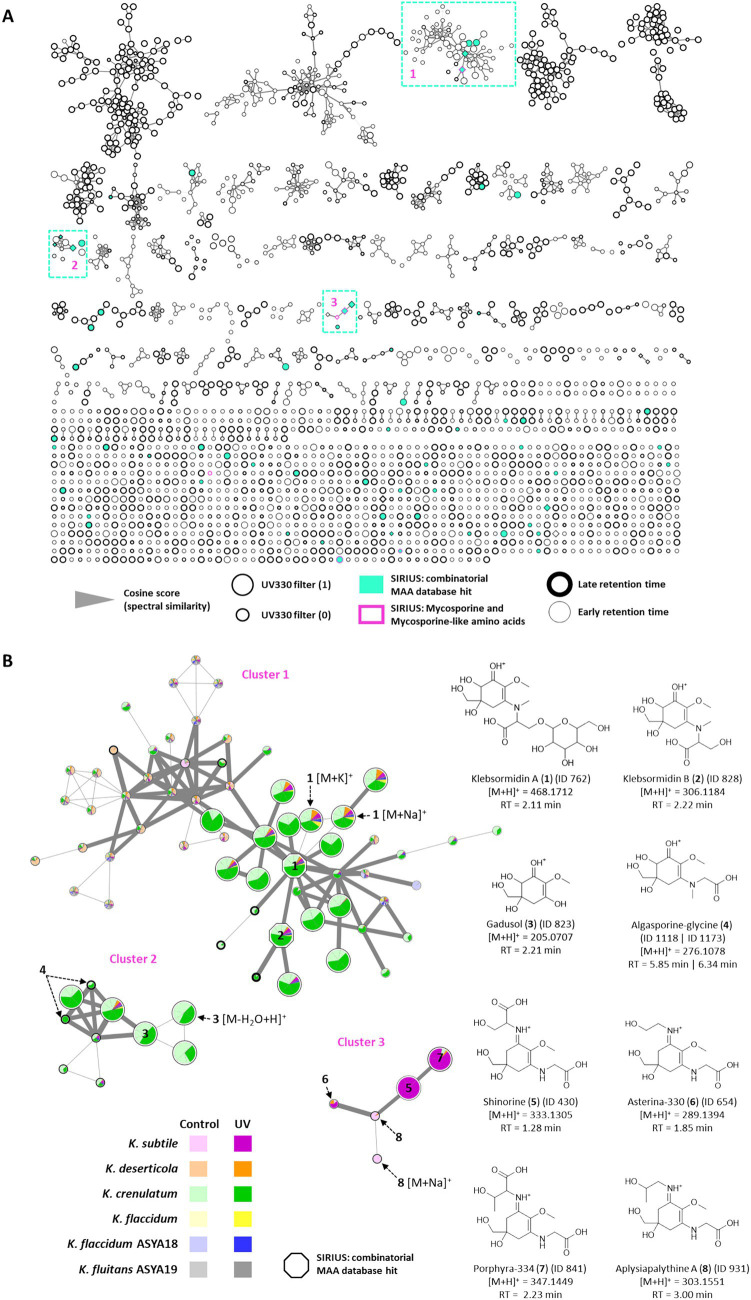
**(A)** Feature-based molecular network generated from the algal extracts. Clusters potentially containing mycosporine-like amino acids (MAAs) are highlighted with cyan boxes and corresponding labels. Nodes annotated as MAAs via SIRIUS are shown with a pink border line. Node size reflects UVR absorption characteristics, with larger nodes indicating features coeluting with a peak at 330 nm (UV330 filter: 1). Node border thickness represents retention time, increasing with later elution. The spectral similarity of two connected nodes is represented by the thickness of the edge. **(B)** Detailed view of MAA-containing clusters. The relative abundance of each feature in the algal extracts is displayed as a pie chart on the respective node. Nodesthat matched entries from the combinatorial MAA database within SIRIUS are represented by octagonal shapes. Putative annotation results are shown on the right, including molecular structures, observed *m/z* values, trivial names, and corresponding retention times. Node size again represents the UV filter variable, with larger nodes indicating absorption at 330 nm. The numbering of annotated compounds corresponds to the labels on the right.

Using the HPLC-DAD method by Hartmann et al. (2020), the major MAAs of *Klebsormidium*, namely klebsormidin A and B, were quantified. For this purpose, porphyra-334, which was available in-house as a pure reference standard and exhibits an absorption spectrum similar to that of klebsormidin A and B, was employed as a surrogate standard. A linear calibration curve was generated based on the peak areas recorded at 320 nm from the analysis of six calibrator solutions (1,000, 100, 50, 10, 5, and 1 μg mL^−1^): a_Area_ = 19.89 x c_concentratio*n*_ + 19.86, R^2^ = 0.9999, LOD = 0.90 μg mL^−1^, LOQ = 2.72 μg mL^−1^. As shown in the chromatogram of *Klebsormidium fluitans* ASYA19 UVR treatment group recorded at a detection wavelength of 320 nm (Figure 7), the retention time of klebsormidin A was 8.90 min, while that of klebsormidin B was 9.60 min. Although trace amounts of MAAs different from klebsormidin A and B were observed in some species with stress-based induction (Figure 8B), they were excluded from the calculation for total accumulated MAA quantification. Interestingly, certain species appeared to maintain constitutive MAA pools or showed differences in MAA composition that may contribute to reducing UVR-induced damage, while others primarily increased MAA production in response to UV-B exposure.

In *K. subtile*, while MAAs were not detected in the control group, a total MAA content of 0.83 mg g^−1^ dry weight (dw) was observed due to UV-B exposure. The same pattern was observed in *K. deserticola*, where the total quantity of MAAs after UV-B treatment was 1.21 mg g^−1^ dw (Figure 6). The response of *K. crenulatum* differed from that of the other species. Control groups already exhibited a high total amount of MAAs (12.97 mg g^−1^ dw), which increased only slightly to 15 mg g^−1^ dw during UV-B treatment. Klebsormidin B was detected after UV-B treatment in some species, but amounts were very low (<0.1 mg g^−1^ dw) in all samples except in *K. crenulatum*. Therefore, klebsormidin A was the main contributor to the total MAA content. In *K. crenulatum*, the MAA content was 0.58 mg g^−1^ dw after UV-B treatment, compared with 0.55 mg g^−1^ dw in the control group, showing no significant difference. In *K. flaccidium*, *K. flaccidium* ASYA18, and *K*. *fluitans* ASYA19, no MAAs were detected in the control groups. After UVR treatment, MAA contents increased to 0.76, 0.32, and 0.25 mg g^−1^ dw, respectively.

Stacked bars show the total MAA content (mg g^−1^ DW) at day 5 (after 72 h UV-B), partitioned into klebsormidin A (blue) and klebsormidin B (orange), quantified by HPLC–DAD. For each species [*K. subtile, K. deserticola, K. crenulatum, K. flaccidum* (Arctic), *K. flaccidum* ASYA18 (Antarctica), and *Klebsormidium fluitans* ASYA19 (Antarctica)], MAA levels for both control (PAR-only; C) and UVR (2 W m^−2^ UV-B + PAR, days 2–5; UV-B) are shown. Bars are means ± SD (*n* = 3 independent biological and technical replicates). Only klebsormidin A and B are plotted; additional trace MAAs detected by UHPLC-HRMS/MS are interpreted in the FBMN analysis.

### Molecular networking-based analysis of the *Klebsormidium* MAA metabolome

After processing the data with MZmine, feature-based molecular networking enabled the organization and visualization of 2,351 nodes, interconnected by 1,948 edges and grouped into 118 clusters with three or more nodes; 1,045 features remained as singletons (Figure 8A). The MAA identification workflow integrated a UV-based filter, since absorption near 330 nm is a characteristic of these compounds, in-house and public spectral library searches, and advanced bioinformatics annotation tools. Altogether, 959 precursor ions, representing roughly 40% of the features, were found to coelute with compounds absorbing at 330 nm, suggesting several high-abundance MAA candidates within the network.

Chemotaxonomic classification using CANOPUS within SIRIUS identified six features (0.3%) in the “mycosporine and mycosporine-like amino acids” (NPC class) category. The ten most represented compound classes in the dataset were “amino acids” (263 features, 12.5%), “phenylethylamines” (113 f., 5.4%),” open-chain polyketides” (91 f., 4.3%), “dipeptides” (90 f., 4.3%), “imidazole alkaloids” (73 f., 3.5%), “cyanogenic glycosides (72 f., 3.4%), “aminosugars” (57 f., 2.7%), “purine alkaloids” (55 f., 2.6%), “pyridine alkaloids” (53 f., 2.5%), and “purine nucleosides” (47 f., 2.2%).

Combining SIRIUS with a recently developed combinatorial MAA database (https://zenodo.org/records/18478940) which leverages a set of known MAA core structures (i.e., gadusol and deoxygadusol) and amino acid-related substituents (with/without *N*-methyl substitution), yielded 60 MAA-related hits, while MS^1^-level local compound database searches in MZmine produced 20 candidates (Figure 8A). Combining all results enabled the putative annotation of eight MAAs or related compounds, such as gadusol, across three clusters (Figure 8B). Cluster 1 included features representing klebsormidin A and B (with potassium and sodium adducts), with UVR-treated *K. crenulatum* samples showing the highest abundances of these compounds. Most UV-active features here were predominant in *K. crenulatum*, suggesting a comparatively diverse MAA-associated feature pattern. Cluster 2 featured nodes mainly from *K. crenulatum*, assigned as gadusol [plus its (M-H_2_O + H)^+^ adduct] and algasporine-glycine, recently described in *Apatococcus ammoniophilus* (https://zenodo.org/records/18478940). Since the node annotated as gadusol had the same retention time as the klebsormidin B feature, in-source fragmentation cannot be ruled out as its origin. In cluster 3, features were annotated as shinorine, asterina-330, porphyra-334, and aplysiapalythine A (including its sodium adduct), and were found to be especially prominent in *K. subtile* samples.

### Cross-metric integration at 72 h: physiology–chemistry links

Growth (*μ*), PSII [Y(II)], and total MAA content (mg g^−1^ dry weight) of *Klebsormidium* species were compared at the end of the 72-h UV-B application, i.e., (day 5) within the 7-day experimental setup (Table 2). A clear decrease in the Y(II) and growth, and a substantial increase in MAA content (+1.45 mg g^−1^) was observed in *K. subtile*, indicating the physiological cost of UVR exposure. *K. deserticola* showed a similar pattern, with increased MAA content (+1.21 mg g^−1^) accompanied by only a small decrease in Y(II) and growth, suggesting inducible MAA accumulation without proportional photochemical recovery. In *K. crenulatum*, the already high constitutive MAA pool increased only slightly (≌ 13 → 15 mg g^−1^), while growth remained unchanged and only moderate suppression of ΔY(II) was observed, consistent with constitutive MAA priming. The polar isolates *K. flaccidum* (Svalbard-Arctic), *K. flaccidum* ASYA18 (Antarctica), and *K*. *fluitans* ASYA19 (Antarctica) showed minimal changes in Y(II) and small Δμ despite comparatively low absolute MAA levels (0.76, 0.33, 0.26 mg g^−1^), suggesting that additional constitutive protective mechanisms may contribute to UV-B tolerance in these species.

**Table 2:** Cross-metric summary at 72 h UV-B exposure across six *Klebsormidium* species.

Species name	μC	μUV	Δμ	Y (II)_C_	Y (II)_UV_	ΔY (II)	MAA_C_	MAA_UV_	ΔMAA (mg g^−1^ dw)	FC_MAA
*Klebsormidium subtile* (Arctic–Alaska; clade E)	0.44	0.38	−0.05	0.53	0.34	−0.19	0.06	1.52	1.45	20.60
*Klebsormidium deserticola* (Chile semi-arid; clade G)	0.30	0.19	−0.11	0.50	0.48	−0.02	0.00	1.21	1.21	121.17
*Klebsormidium crenulatum* (Alps; clade F)	0.44	0.45	0.00	0.51	0.42	−0.08	12.98	15.02	2.04	1.16
*Klebsormidium flaccidium* (Arctic–Svalbard; clade B\C)	0.59	0.57	−0.02	0.62	0.57	−0.04	0.00	0.76	0.76	53.25
*Klebsormidium flaccidium* ASYA18 (Antarctica; clade B\C)	0.45	0.49	0.04	0.58	0.57	0.00	0.00	0.33	0.33	32.89
*Klebsormidium fluitans* ASYA19 (Antarctica; clade E)	0.64	0.63	−0.01	0.59	0.53	−0.05	0.00	0.26	0.26	25.71

Values are means (*n* = 3) for each species and treatment. μC and μUV are specific growth rates (d^−1^) calculated from chlorophyll-fluorescence–based growth (ln Ft/F0)/t over the experimental window ending at day 5; Δμ = μUV − μC (negative values indicate UV-inhibition). Y(II)_C_ and Y(II)_UV_ are the effective PSII quantum yields measured by PAM at day 5; ΔY(II) = Y(II)_UV_ − Y(II)_C_. MAA_C_ and MAA_UV_ are total mycosporine-like amino acids (mg g^−1^ DW; sum of klebsormidin A + B by HPLC-DAD) quantified from biomass harvested at day 5; ΔMAA = MAA_UV_ − MAA_C_. FC_MAA is the fold-change in total MAAs under UV relative to control (MAA_UV_\MAA_C_).

To avoid pseudoreplication, one value per species was used for correlation analyses (*n* = 6). Spearman’s rank correlation at day 5 revealed contrasting trends consistent with different MAA deployment strategies. Species with higher baseline MAA levels (measured in control samples at day 5) tended to experience smaller Y(II) decreases under UV-B exposure, as suggested by a negative trend with ΔY(II) (*ρ* = −0.78; p ≈ 0.06–0.08). In contrast, MAA fold change showed a positive trend with ΔY(II) (*ρ* = +0.77; p ≈ 0.07–0.08), where species with low constitutive MAA levels but strong inducible increases also displayed larger declines in Y(II). This pattern is consistent with contrasting constitutive inducible photoprotective responses rather than with total MAA quantity alone. Relationships between Y(II) decline and total MAA levels under UV-B (MAA_UV_) or ΔMAA were weaker and not statistically significant. Growth responses were also not clearly associated with MAA levels or MAA changes. Overall, these results suggest that photochemical costs are more closely associated with MAA deployment strategy (pre-existing versus induced) than with total MAA content alone. Results of within-species UV-B effects on growth and Y(II) are summarized in Table 2.

## Discussion

### Phylogenetic diversity and ecological context of study species

The distribution of the six species across four distinct *Klebsormidium* clades indicates that this genus has adapted to a wide range of habitats during its evolutionary history. The polar species in the B/C clade (*K. flaccidum*, *K. flaccidum* ASYA18) comprise species capable of living on limestone surfaces and exhibiting filamentous structures morphologically consistent with previous studies (Mikhailyuk et al., 2014; Glaser et al., 2017). The cosmopolitan nature of the E clade includes species with a broad adaptability to terrestrial habitats, such as *Klebsormidium fluitans* ASYA19 and *K. subtile* (Rindi et al., 2008; Sluiman et al., 2008). The alpine-origin *K. crenulatum* (F clade) and the arid-region species *K. deserticola* (G clade) further expand this ecological diversity (Rindi et al., 2011; Samolov et al., 2019). Consequently, the distinct divergence in the responses of these species from different clades to UV-B stress indicates that both genetic origin and habitat conditions shape photoprotection strategies.

### Growth performance under UV-B: interpretation of species-specific responses

Many *Klebsormidium* members are distributed across manifold habitats worldwide, including extreme environments such as Antarctica (Rindi et al., 2008). While recent studies have investigated the effects of various abiotic stressors on *Klebsormidium*, assessing both physiological and metabolic consequences, the effects of UVR are still understudied (Palaniappan et al., 2025; Kolomiiets et al., 2025). Growth rate is a common proxy to characterize the physiological performance of microalgae under stress conditions, providing an easy-to-measure integrative indicator of how metabolic processes are positively or negatively affected by the stressors. To date, only one study has investigated the consequences of UVR exposure on MAA accumulation and growth rate simultaneously in *Klebsormidium*, focusing on only *K. fluitans*. The data showed that increased UV-B exposure moderately suppressed the growth rate in isolates sampled from 5 different altitudes (from ~ 0.7 *μ* d^−1^ in controls to ~ 0.5 μ d^−1^) (Kitzing et al., 2014). For comparison, the growth rate of *K. crenulatum* in the present study was 0.3 μ d^−1^, while *K. dissectum* grew with 0.5 μ d^−1^ (Karsten et al., 2010; Karsten and Holzinger, 2012). This suggests that *Klebsormidium* species prioritize different metabolic defense mechanisms besides growth to cope with stress. UV-B treatment did not arrest growth in any of the *Klebsormidium* species investigated in the present study, but rather caused partial temporary suppressions. This decrease in growth was species-specific and might reflect different genetic backgrounds or adaptive traits. Interestingly, no growth suppression was found in species from the Arctic, Antarctica, and the Alps. In contrast, both the Alaskan and Chilean semi-arid species, *K. subtile* and *K. deserticola,* showed temporary suppression. Notably, *K. deserticola* did not restore growth to control levels. As the only species originating from a high-temperature, semi-arid environment, this suggests a potential role of environmental adaptation in the observed lack of recovery. Previous studies on Polar species showed that growth due to UV-B exposure is usually unaffected or only temporarily reduced (Robinson et al., 2024). Research on algal communities in Antarctic glaciers, based on chlorophyll *a* concentration, suggests that algae protect themselves from UV-B radiation using several strategies. One of the best-known protective mechanisms is the production of MAAs (Ryan et al., 2012).

Overall, UV-B exposure resulted in a species-specific suppression of growth rates in *Klebsormidium*. *K. deserticola* showed higher vulnerability, indicating that it possesses a lower tolerance to continuous UV-B exposure. It is worth noting that it is the only species not isolated from extremely low temperature environments such as the Arctic, Alpine, and Antarctic. The Alpine *K. crenulatum* was significantly impacted during the exposure but restored its growth rate, as seen from the *K. subtile*. Taken together, these results show that the Alpine, Arctic, and Antarctic species exhibit a higher tolerance to continuous UV-B exposure.

### PSII efficiency under UV-B: photochemical responses

Photosynthesis plays a key role in metabolism and is an important marker of the cell’s physiological state in response to stress (Kruse et al., 2025). It can be measured non-invasively via PAM fluorimetry, where the Y(II) serves as a widely used proxy for the photosynthetic performance (Klughammer and Schreiber, 2008). Kitzing et al. (2014) found a minor decline in Y(II) under increasing UV-B exposure in *K. fluitans*. A subsequent study by the same authors investigated the consequences of UV-B for the optimum quantum yield of PSII (F_v_/F_m_) in 32 species of *Klebsormidium* and its sister genera *Entransia*, *Hormidiella*, and *Interfilum* (Kitzing and Karsten, 2015). The comparison revealed that most *Klebsormidium* species showed only minimal F_v_/F_m_ changes, while *Entransia* was strongly inhibited. This indicates that terrestrial filamentous microalgae are well adapted to UVR, reflecting their habitat conditions. On the other hand, UVR exposure has severe effects on the photosynthesis of aquatic algae (Karsten, 2008).

In the present study, *K. subtile* showed a stronger decline in photosynthesis after UVR exposure than the other species. This indicates that they are more susceptible to UVR, but are still able to recover within a relatively short timeframe. Recent work suggests that this recovery occurs when multiple photoprotective mechanisms are activated at the same time, triggering cellular repair and protection (Görünmek et al., 2024). Overall, these results show that UVR tolerance in terrestrial microalgae is species-specific and influenced by factors such as geographic origin (low vs. high latitude), genetic background, and season. Differences in photosynthetic responses among geographic isolates of the same genus may result from the rapid action of sunscreen compounds, which are the focus of this study, or from additional protective mechanisms such as carotenoids, DNA repair processes, potentially flavonoids, and other cellular responses, as reported in earlier studies (Pessoa, 2012).

Additionally, growth rate (*μ*) and effective quantum yield of PSII (Y(II)) did not always respond in parallel across species, indicating that short-term photochemical stress and longer-term biomass accumulation are not necessarily directly coupled under UV-B exposure. Y(II) reflects the immediate functional state of PSII and is therefore highly sensitive to transient photoinhibition, whereas fluorescence-derived growth rate integrates biomass development over time and may reflect compensatory recovery processes (Maxwell and Johnson, 2000; Baker, 2008; Albrecht et al., 2022). Consequently, temporary reductions in Y(II) do not always result in proportional growth inhibition if repair mechanisms, constitutive protection, or acclimatory responses maintain overall physiological performance. In addition, because chlorophyll fluorescence was used as a proxy for growth, fluorescence-based growth estimates under UV-B exposure may partly reflect short-term photophysiological responses in addition to biomass accumulation, which should be considered when interpreting species-specific differences.

### Constitutive and inducible photoprotection strategies across *Klebsormidium* clades

We suggest two different MAA-based protection strategies employed by *Klebsormidium* to cope with UV-B exposure. First, “constitutive MAA priming”, which is utilized by *K. crenulatum*, shows high klebsormidin A and minor klebsormidin B concentrations under PAR-only conditions. Upon UV-B exposure, this MAA pool increases slightly. We suggest that this strategy is ideal for coping with fluctuation and rapidly onsetting UV-B exposures, minimizing the need for *de novo* MAA synthesis. This idea is also consistent with the observation that certain aeroterrestrial streptophyte algae always possess high MAA pools as a safeguard against sudden exposure to UVR or desiccation, without experiencing UVR before (Joshi et al., 2018; Hartmann et al., 2020). Moreover, this MAA pool contains the MAA precursor gadusol, which has an additional hydroxyl group at the carbon-6 position of the core structure, the starting point of MAA synthesis in certain Chlorophyta (Hartmann et al., 2016). The second MAA-based strategy is employed by species such as *K. subtile* and *K. deserticola*, which show MAA pools under PAR below the detection limit, but a strong increase when exposed to UV-B. This suggests that MAAs in these species have their major role in providing UVR tolerance, but no role under UVR-limited conditions. FBMN analyses showed that cellular stress, in particular UVR stress, can not only increase MAA accumulation but also change the spectral coverage, increase the antioxidant effect, and or cellular compartmentalization. This type of metabolic response advocates that in terrestrial taxa, PSII penalties during increased MAA accumulation support the organism to maintain itself in the recovery phase without causing complete suppression of the photosystem due to UV-B (Kitzing and Karsten, 2015).

Moreover, a recent study showed that terrestrial filamentous green algae benefit from vertical growth within the polysaccharide-rich biofilms they form. Because these epiphytic biofilms often develop on tree surfaces exposed to strong light, the algae require additional protection, such as mycosporine-like amino acids (MAAs) (Søchting et al., 2025).

Taken together, these findings confirm three distinct strategies for UV-B protection in *Klebsormidium*: an inducible MAA response in UVR-sensitive species (*K. subtile*, *K. deserticola*), non-MAA-dependent constitutive protection in polar species, and MAA pre-priming in *K. crenulatum*. These strategies reflect the classic trade-offs between constitutive and inducible defenses in stress biology and show that UVR tolerance depends on composition, timing, and strategy rather than total MAA levels alone (Hartmann et al., 2020; Rastogi et al., 2010).

When the values are considered together with the structural MAA pool observed in *K. crenulatum* and the compositional change caused by UVR, these patterns reveal that *Klebsormidium* members exhibit a strategic segmentation from constitutive MAA supplying to adaptive accumulation. This suggests that exposure of cells to UVR in their natural environment can lead to predictable changes that enhance their biosynthetic capacity, thus preparing their metabolism for impending conditional alterations.

### FBMN reveals species-specific MAA composition and hidden metabolomic diversity

FBMN analysis of the extracts revealed several features (i.e., precursor ions eluting at specific retention times) that could be assigned to the chemotaxonomic class of MAA. Using a combinatorial MAA database, a subset of these features could be putatively annotated; however, confident annotations were obtained for only a limited number of nodes. Because only klebsormidin A and B were quantitatively assessed using available standards, additional UV-absorbing compounds detected through FBMN analyses should be regarded as putatively annotated features pending future targeted structural confirmation.

In Cluster 1, which contained the species-specific MAAs klebsormidin A and B, several UV_330_-absorbing features remained unannotated. This observation strongly suggests the presence of additional, currently unknown MAAs in *Klebsormidium* species. The occurrence of such uncharacterized UV-active compounds is consistent with the pronounced chemical diversity reported also for other algal MAA profiles and indicates that the MAA biosynthetic capacity of *Klebsormidium* may be more diverse than currently recognized.

Cluster 2 contained gadusol as well as the recently discovered algasporin-glycine compound, which is now being identified for the first time in a streptophyte algal species (Hammerle et al., n.d.). This is plausible from a biosynthetic perspective, as gadusol represents a central precursor in MAA biosynthesis, while algasporine-glycine, bearing a characteristic *N*-methyl substitution, is structurally related to the klebsormidins A and B.

Notably, in UVR-treated *K. subtile* samples, additionally, annotations for the canonical/standard MAAs shinorine, asterina-330, porphyra-334, and aplysiapalythine A were obtained. This finding is unexpected, as these compounds bear a deoxygadusol core structure and are thus structurally distinct from the klebsormidins. The presence of both klebsormidin-type and canonical MAAs raises the question of whether the genus *Klebsormidium* harbors multiple, partially independent biosynthetic pathways for MAA production, or whether UVR exposure preferentially induces the biosynthesis of standard MAAs over that of the species-specific klebsormidins.

### Integrating physiology and chemistry: linking growth, PSII performance, and MAA dynamics

Analysis of all results after 72 h of UV-B exposure revealed a consistent but non-linear relationship between biochemical and physiological responses among the species. For example, *K. crenulatum* possessed a large constitutive MAA pool in the control group and showed only a modest increase after UV-B exposure, while growth (Δ*μ* ≈ 0) and PSII efficiency [ΔY(II) ≈ −0.08] remained relatively unchanged. In contrast, other species (*K. subtile, K. deserticola, K. flaccidum, K. flaccidum* ASYA18, and *K. fluitans* ASYA19) started from very low or undetectable MAA concentrations and displayed stronger MAA induction after UV-B exposure. However, the physiological responses of these species were variable [Δμ between −0.11 and +0.04; ΔY(II) between −0.19 and 0.00]. Taken together, growth and photosynthetic performance reveal distinct patterns of UV-B tolerance among the species. These differences suggest the presence of species-specific protective mechanisms that are not fully explained by total MAA content alone.

**Figure 9:**
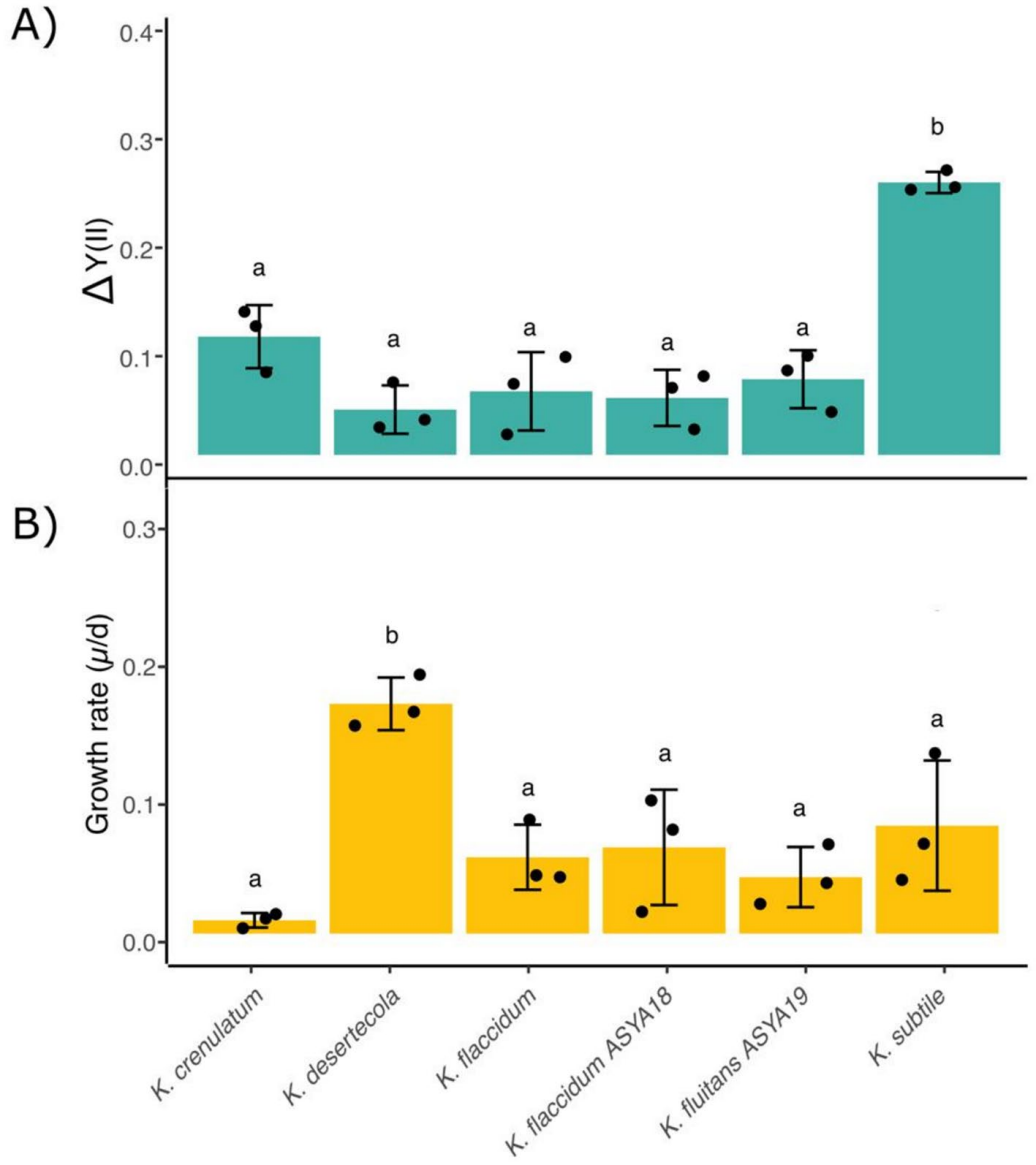
Cross-metric representation of ΔY(II) and Growth rate (μ/d) under UV exposure. **(A)** Difference in effective quantum yield of Y(II) under UV-B radiation compared to control light conditions in *Klebsormidium* species on day 5. **(B)** Difference in growth rate between UV-B–exposed and non-exposed *Klebsormidium* species on day 5. Error bars represent the standard error. Different letters indicate statistically significant differences among treatments as determined by ANOVA (*p*-value <0.05), identical letters indicate no significant difference.

For instance, *K. deserticola*, originating from a semi-arid region, exhibited the strongest reduction in growth rate but maintained relatively stable photosynthetic yield (Figures 9A,B). In contrast, *K. subtile* showed only slight growth inhibition but a more pronounced reduction in Y(II) (Figures 9A, B). These contrasting responses indicate that photochemical stress and growth inhibition are not always directly coupled and may reflect different acclimation strategies depending on species origin and physiology.

MAAs likely function as important UVR protectants but may not provide complete protection alone, suggesting that additional mechanisms such as DNA repair, ROS scavenging, antioxidant systems, and structural cell wall components may also contribute to UV-B tolerance (Shick and Dunlap, 2002; Cockell and Knowland, 1999; Rastogi et al., 2010). This is particularly relevant for polar species, which maintained relatively high UV tolerance despite comparatively low MAA pools. At this stage, however, the specific contribution of these mechanisms remains hypothetical and was not directly measured in the present study.

The lack of a direct positive correlation between Δμ and ΔY(II) further supports this interpretation. For example, *K. subtile* showed strong suppression of Y(II) but only a slight decrease in growth rate. This reinforces the idea that Y(II) reflects the immediate functional state of PSII and is therefore highly sensitive to transient photoinhibition, whereas μ integrates biomass development, resource allocation, and recovery processes over time (Murchie and Lawson, 2013).

## Conclusion

This study shows that UV-B photoprotection in *Klebsormidium* is not based on a single mechanism, but involves distinct protective strategies across phylogenetically different clades. The differentiated responses demonstrate that UVR tolerance is associated with geographical origin, genetic background, habitat conditions, and species-specific metabolic response patterns. MAA quantification together with UHPLC-VWD-HRMS/MS-based untargeted metabolomic analyses suggests three broadly contrasting photoprotective response patterns in *Klebsormidium*. *K. crenulatum* exhibited a constitutive MAA-associated response pattern by maintaining a high basal pool of klebsormidin A and other trace MAA compounds independent of UV-B exposure, providing efficient protection against sudden or fluctuating UVR stress. Species such as *K. subtile* and *K. deserticola* exhibited inducible MAA accumulation responses, rapidly increasing MAA levels in response to UV-B exposure, although being associated with temporary physiological costs. In contrast, polar species such as *K. flaccidum* (Arctic), *K. flaccidum* ASYA18, and *Klebsormidium fluitans* ASYA19 maintained high UVR tolerance despite comparatively low MAA levels, suggesting that additional constitutive protective mechanisms, such as carotenoids, antioxidant systems, or efficient repair processes, may contribute to photoprotection in these species.

Feature-based molecular networking (FBMN) further allowed the putative annotation of several UV-absorbing compounds in *K. crenulatum*, including algasporin-glycine, which was recently reported in the chlorophyte *Apatococcus* and has not previously been described in streptophyte algae, as well as the occurrence of canonical MAAs such as shinorine, asterina-330, and porphyra-334 in *K. subtile*. These findings suggest that MAA-associated chemical diversity in *Klebsormidium* may be broader than currently recognized. The integration of physiological and biochemical data further suggests that UVR tolerance is more closely associated with the composition, timing, and deployment strategy of MAAs than with total MAA content alone. While high basal MAA levels may reduce immediate photochemical damage, inducible MAA accumulation appears to support compensatory stress responses after UV-B exposure. Together, these results highlight that both constitutive protection and inducible acclimation contribute to UV-B resilience in early-diverging streptophyte algae.

## Data Availability

The datasets presented in this study can be found in online repositories. The names of the repository/repositories and accession number(s) can be found in the article/Supplementary material.
