# Supplementary material for: Physiology and chemistry integration under UV-B across green algal Klebsormidium clades (Streptophyta) reveals constitutive and inducible MAA-associated photoprotective responses
Source: Front Microbiol. 2026 Jun 15;17:1858620. doi: 10.3389/fmicb.2026.1858620 (PMC13312787; doi:10.3389/fmicb.2026.1858620)
Supplement: Supplementary file 1 [file Table_1.DOCX]

**Method**

**MS data preprocessing**

The data obtained in positive ionization mode were converted from the standard data format .raw (Thermo Scientific) to the open format .mzML using the MSConvert software included in the ProteoWizard package (Chambers et al., 2012). The converted files were processed with mzmine 4.3.0 (mzio GmbH, Bremen, Germany) (Schmid et al., 2023). A batch file with following specified steps was created: The generated .mzML files were imported with the standard import function. For mass detection at the MS^1^ level, the noise level was set to 5.0E^5^. For MS^2^ detection, the noise level was set to 0.0E^0^. Parameters for the ADAP chromatogram builder module were set as follows: minimum consecutive scans, 5; minimum intensity for consecutive scans, 5.0E^5^; minimum absolute height, 5.0E^5^; scan-to-scan accuracy (*m/z*) of 0.0030 or 10.0 ppm. The Savitzky-Golay algorithm was used for smoothing, with retention time width (scans) set to 5. Chromatogram deconvolution was done with the Local minimum feature resolver (MS/MS scan pairing, allowed; MS^1^ to MS^2^ precursor tolerance, 0.0100 *m/z* or 10.0 ppm; retention time filter, use feature edges; minimum relative feature height, 25.0%; minimum required signals, 1; dimension, retention time; chromatographic threshold, 53.8%; minimum search range RT, 0.200; minimum relative height, 0.0%; minimum absolute height, 5.0E^5^; min ratio of peak top/edge, 2.00; peak duration range, 0.00-6.01; minimum scans, 5). Isotopes were detected with the ^13^C isotope filter (*m/z* tolerance, 0.0010 *m/z* or 3.0 ppm; RT tolerance, 0.08 min; maximum charge, 2; representative isotope, most intense). Isotope signals for H, C, N, O, and S were searched using the isotopic peaks finder (*m/z* tolerance, 0.0010 *m/z* or 3.0 ppm; maximum charge of isotope *m/z*, 1; search in scans, single most intense). An aligned feature list was generated employing the join aligner algorithm (m/z tolerance, 0.0010 *m/z* or 5.0 ppm; weight for *m/z*, 3; retention time tolerance, 0.35 min; weight for RT, 1). The Feature list rows filter was used with the validate ^13^C isotope pattern function allowed (*m/z* tolerance, 0.0010 *m/z* or 3.0 ppm; max charge, 2; estimate minimum carbon, allowed; remove if ^13^C, allowed; exclude isotopes, O). Gap filling was performed with the peak finder (multithreaded) algorithm with the intensity tolerance, *m/z* tolerance (sample-to-sample), retention time tolerance, and minimum scans (data points) set to 20.0%, 0.0030 *m/z* or 10.0 ppm, 0.35 min, and 3, respectively. Duplicates were filtered using the duplicate peak filter module (filter mode, new average), with the m/z tolerance set to 0.0005 *m/z* or 1.5 ppm and the RT tolerance set to 0.14 min. The Feature list rows filter was again applied to reset the feature IDs and retain only features with associated MS² spectra.

The resulting filtered list was subjected to ion identity molecular networking (Schmid et al., 2021), starting with the metaCorrelate module (RT tolerance, 0.11 min; minimum feature height, 0.0E^0^; intensity threshold for correlation, 5.0E^5^; feature shape correlation and feature height correlation, both allowed). Subsequently the ion identity networking algorithm was used (*m/z* tolerance, 0.0010 *m/z* or 3.0 ppm; check, one feature; minimum height, 0.0E^0^; ion identity library: maximum charge, 2; maximum molecules/cluster, 2; adducts, [M+H-H_2_0]^+^, [M+H]^+^, [M+NH_4_]^+^, [M+Na]^+^, [M+K]^+^, [M-H+2Na]^+^, [M+2H]^2+^; annotation refinement: delete small networks without major ion, yes; delete smaller networks, link threshold, 4; delete networks without monomer, yes). Feature annotations were obtained with the local compound database search (database of MAAs) as well as with the lipid annotation module. Lastly, the aligned feature list was exported with the dedicated functions for molecular networking and for analysis with SIRIUS.

**Feature-based molecular network generation**

A molecular network was created with the feature-based molecular networking (FBMN) workflow on the GNPS2 webpage (https://gnps2.org/homepage) (Nothias et al., 2020): The mass spectrometry data were first processed with mzmine 4.3.0 (see section above), and the results were exported to GNPS2 for FBMN analysis. The precursor ion mass tolerance was set to 0.02 Da, and the MS/MS fragment ion tolerance to 0.02 Da. Both the window filter as well as the precursor window filter were allowed. A molecular network was then created where edges were filtered to have a cosine score above 0.6 and more than 5 matched peaks. Further, edges between two nodes were kept in the network if and only if each of the nodes appeared in each other’s respective top 10 most similar nodes. Finally, the maximum size of a molecular family was set to 100, and the lowest-scoring edges were removed from molecular families until the molecular family size was below this threshold. The spectra in the network were then searched against GNPS spectral libraries (Wohlgemuth et al, 2016; Horai et al., 2010). The library spectra were filtered in the same manner as the input data. All matches kept between network spectra and library spectra were required to have a score above 0.6 and at least 4 matched peaks. Additional edges were provided as well. The molecular networks were visualized using Cytoscape software (Shannon et al., 2003). The feature-based molecular networking job can be publicly accessed at: https://gnps2.org/status?task=aa3139f533744e578c11464ecb169982#.

**MAA filter variable**

A Cytoscape-compatible information layer highlighting features in the network capable of absorbing light of 330 nm was prepared using a dedicated workflow (Hammerle et al. 2021, 2023). The UV trace of each extract investigated was extracted and visualized as a chromatogram with OriginPro 2020 (OriginLab Corporation, Northampton, Massachusetts, USA). Chromatograms were integrated to obtain peaks with start and end points. The threshold value was set to 5000 mAU. Excel 365 was then used to determine, for each feature in the combined feature list, in which extract that specific feature was present with the largest peak area. The retention times of the UV peaks were then matched with those of the features (MS peaks). The dead volume between the DAD and MS detector was negligible. If the feature could be assigned to a peak, the value was set to 1. If it could not be assigned, the value of this variable was set to 0. The results were exported as a .csv file.

**SIRIUS metabolite annotation**

The corresponding .mgf file exported from mzmine 4.3.0 was processed with SIRIUS 6.1.1 (Dührkop et al., 2019). The parameters were set as follows: instrument, Orbitrap; MS^2^ mass accuracy, 10 ppm; fix formula for detected lipid, yes; fallback adducts: [M+H]^+^, [M+Na]^+^, [M+K]^+^; molecular formula generation, de novo + bottom up. The prediction of fingerprints was carried out with CSI:FingerID and the prediction of chemical classes with CANOPUS (Dührkop et al., 2015, 2021). Searches for molecular formulas and structures were limited to the combinatorial MAA database (Hammerle et al., 2025) to exclusively obtain MAA-specific annotations.

**Explanation of annotation levels**

According to the Metabolomics Standards Initiative (MSI; Sumner et al., 2007), annotation level 1 refers to identified compounds confirmed by matching both LC retention time and full MS/MS data with authentic standards. Annotation level 2 corresponds to putatively annotated compounds that lack chemical reference standards but show spectral similarity to entries in spectral libraries. Annotation level 3 denotes putatively characterized compound classes, while annotation level 4 includes unknown compounds that can be distinguished and quantified but lack structural information.

**Results**

**
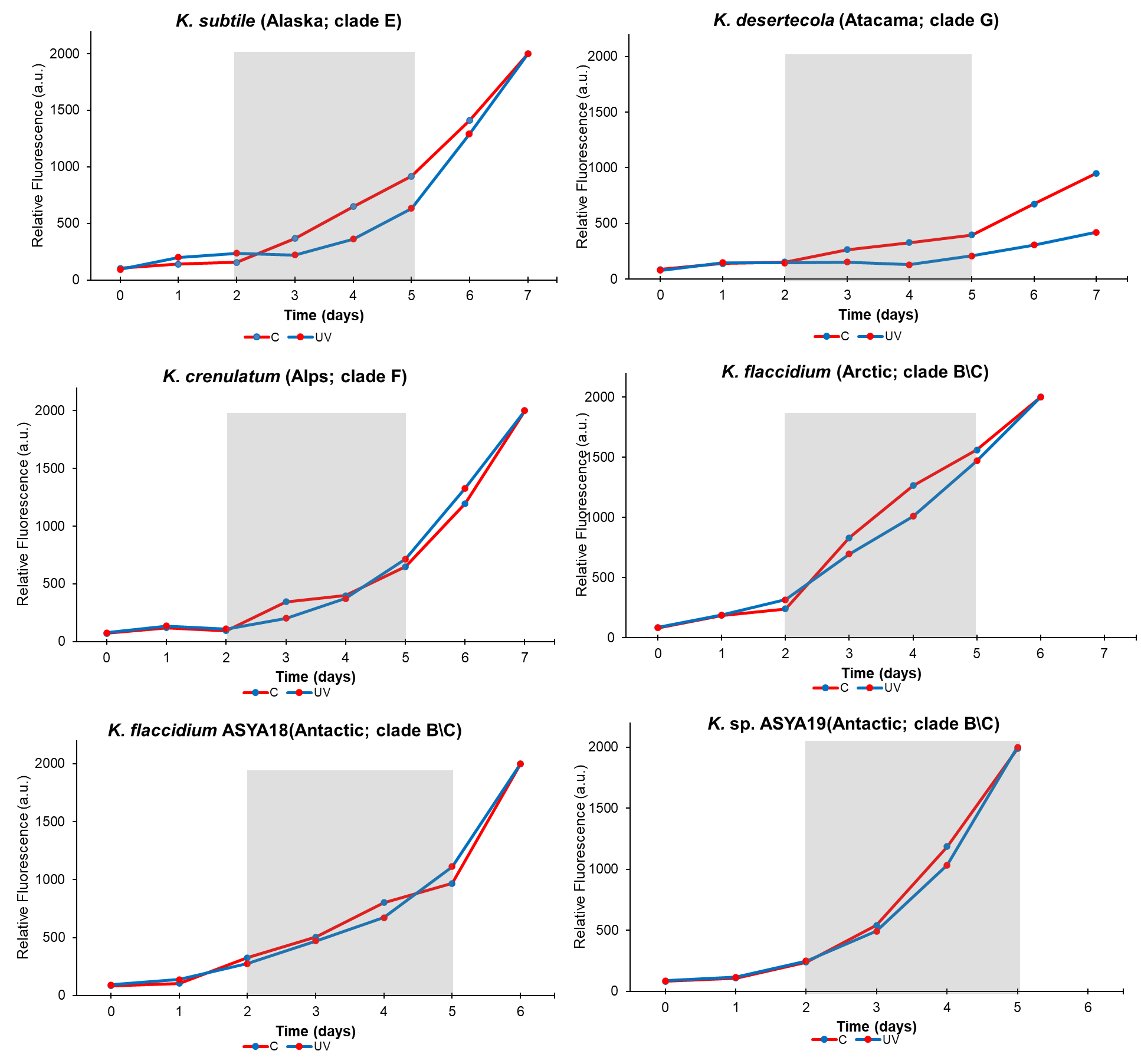
**

**Supplementary Figure S1** Effect of UV-B exposure on growth in six *Klebsormidium* strains.
Time series of relative chlorophyll fluorescence (a.u.) as a proxy for biomass for control (red) and UV-B–treated (blue) cultures over 7 days. Panels show strains from distinct regions/clades: *K. subtile*, *K. deserticola*, *K. crenulatum*, *K. flaccidum*, *K. flaccidum* ASYA18, and *Klebsormidium* sp. ASYA19. Cultures were maintained under PAR = 50 µmol photons m⁻² s⁻¹; gray shading denotes the UV-B induction window (days 2–5; 2 W m⁻² UV-B + PAR) bracketed by PAR-only pre-acclimation (days 0–2) and PAR-only recovery (days 5–7). Fluorescence was recorded daily with a blue-LED (470 nm) growth fluorometer; curves show the mean (±SD) of n = 3 biological replicates.

**
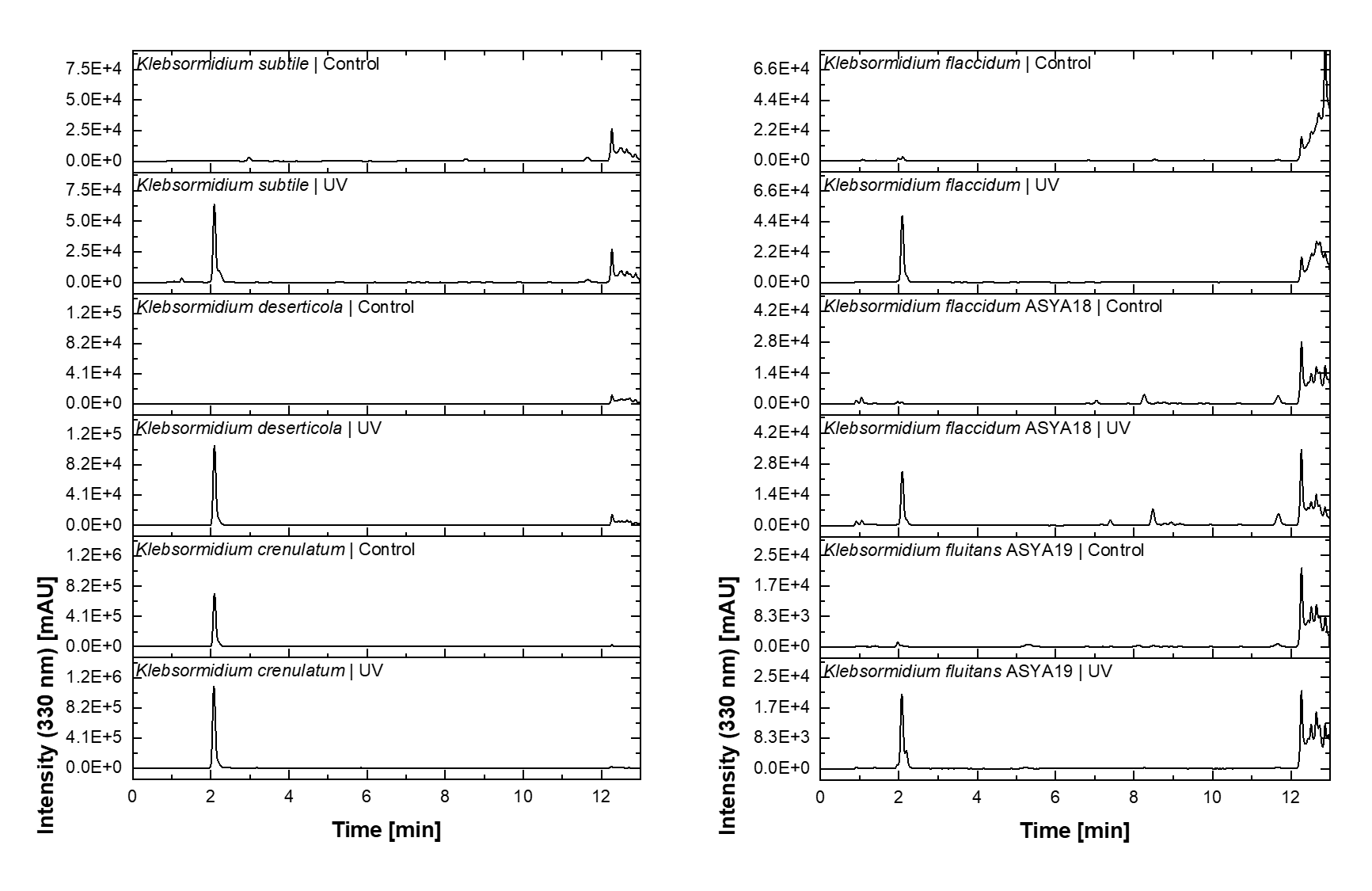
Supplementary Figure S2** Representative UHPLC–VWD chromatogram (330 nm) of *Klebsormidium* extracts.

UHPLC–VWD trace recorded at λ = 330 nm (MAA absorption maximum) showing the MAA region of the extract (strain/treatment indicated in panel title). Identification of klebsormidin A (RT_HPLC_ ≈ 8.6–9.2 min) and klebsormidin B (RT_HPLC_ ≈ 9.4–10.1 min) was supported by UV spectra (290–400 nm) and MS/MS (see Methods). Baseline and scaling are in mAU; minor peaks correspond to low-abundance co-eluting metabolites detected by HRMS but below quantitation by VWD.


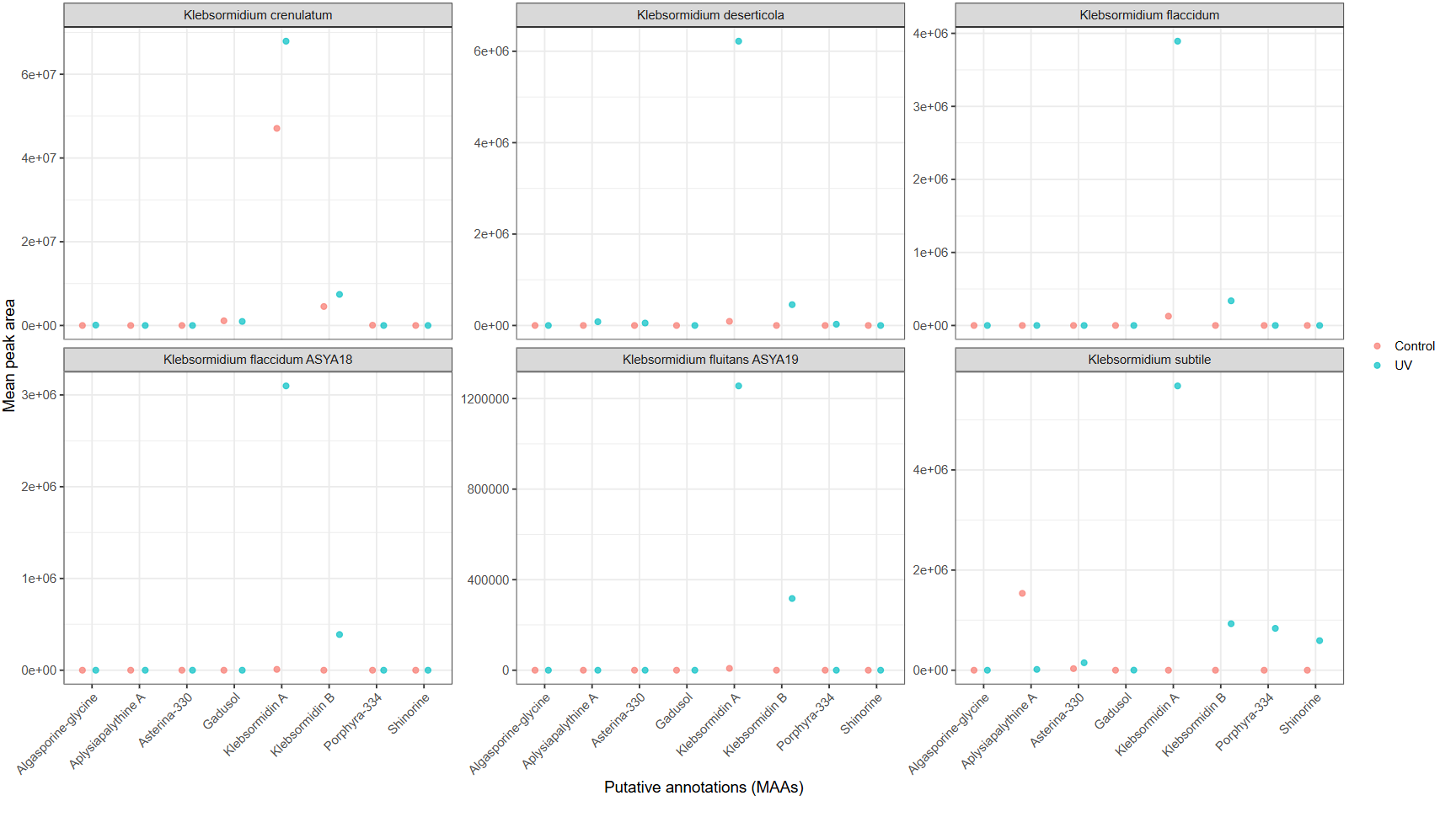


**Supplementary Figure S3** Composition-resolved MAA profiles across six *Klebsormidium* strains (UHPLC-HRMS/MS).

Dot plots show mean peak area (arbitrary MS units) for putatively annotated MAAs in control (red) and UV-B (blue) cultures at the 72-h endpoint. Panels correspond to strains: *K. flaccidium* ASYA-18 (Antarctica), *K.* sp. ASYA-19 (Antarctica), K. crenulatum (Alps), K. deserticola (Atacama), K. flaccidum (Arctic), and K. *subtile.* Analytes include klebsormidin A, klebsormidin B, gadusol, asterina-330, porphyra-334, shinorine, aplysiapalythine A, and algasporine-glycine. Values derive from XIC-integrated features. Annotations are supported by MS/MS similarity (FBMN/GNPS), retention time/UV spectra, and are reported with Schymanski confidence levels in Methods. Note that low-abundance compounds visible here may fall below HPLC–DAD quantitation and are interpreted in the molecular-network analysis.

List of putative annotations

Table S1. Annotation results combining the output of the in-house database search (mzmine), GNPS library hits, and the chemotaxonomic classification as well as the structure suggestions obtained through SIRIUS.

|  | # | Feature ID | Retention time [min] | Precursor m/z | Adduct | Precursor formula | GNPS: Library results | SIRIUS: Median mass error | SIRIUS: NPC#class | SIRIUS: Annotations | In-house database: Annotations | In-house database: SMILES codes | Final annotation (Annotation level) |
| --- | --- | --- | --- | --- | --- | --- | --- | --- | --- | --- | --- | --- | --- |
| Cluster 1 | 1 | 762 | 2.11 | 468.1712 | [M+H]^+^ | C18H29NO13 |  | 5.23 | Cyanogenic glycosides | Klebsormidin A | Klebsormidin A | O=C1C(O)C(O)(CO)CC(N(C)C(C(O)=O)COC2OC(CO)C(O)C(O)C2O)=C1OC | Klebsormidin A (1) |
|  | 2 | 828 | 2.22 | 306.1184 | [M+H]^+^ | C12H20NO8 |  | 5.37 | Mycosporine and Mycosporine-like amino acids | Klebsormidin B | Klebsormidin B | O=C1C(O)C(O)(CO)CC(N(C)C(C(O)=O)CO)=C1OC | Klebsormidin B (1) |
| Cluster 2 | 3 | 823 | 2.21 | 303.1551 | [M+H]^+^ | C8H13O6 |  | 5.57 | 2-pyrone derivatives | Gadusol | Gadusol | OC(CC(CO)(O)C1O)=C(OC)C1=O | Gadusol (2) |
|  | 4 | 1118 | 5.85 | 276.1078 | [M+H]^+^ | C11H18NO7 |  | 3.35 | Cyanogenic glycosides | Algasporine-glycine | Algasporine-glycine | OC(CO)(C(O)C1=O)CC(N(C)CC(O)=O)=C1OC | Algasporine-glycine (2) |
| Cluster 3 | 5 | 430 | 1.28 | 333.1305 | [M+H]^+^ | C13H21N2O8 | Shinorine | 4.22 | Mycosporine and Mycosporine-like amino acids | Shinorine | Shinorine | OC(CC(NCC(O)=O)=C/1OC)(CO)CC1=N/C(C(O)=O)CO | Shinorine (1) |
|  | 6 | 654 | 1.85 | 289.1394 | [M+H]^+^ | C12H21N2O6 |  | 4.43 | Mycosporine and Mycosporine-like amino acids | Asterina-330 | Asterina-330 | OC(CC(NCC(O)=O)=C/1OC)(CO)CC1=N/CCO | Asterina-330 (1) |
|  | 7 | 841 | 2.23 | 347.1449 | [M+H]^+^ | C14H23N2O8 | Porphyra-334 | 4.98 | Cyanogenic glycosides | Porphyra-334 | Porphyra-334 | OC(CC(NC(C(O)C)C(O)=O)=C/1OC)(CO)CC1=N/CC(O)=O | Porphyra-334 (1) |
|  | 8 | 931 | 3.00 | 303.1551 | [M+H]^+^ | C13H23N2O6 | Palythinol | 4.06 | Mycosporine and Mycosporine-like amino acids | Aplysiapalythine A | Aplysiapalythine A | OC(CC(NCC(O)=O)=C/1OC)(CO)CC1=N/CC(C)O | Aplysiapalythine A (1) |
